# Unveiling Electric Field-Driven Stereocontrol in Hurd–Claisen Rearrangements

**DOI:** 10.1021/acs.jctc.6c00732

**Published:** 2026-06-25

**Authors:** Mateus Rodrigues Barbosa, Pedro H. F. Matias, Daniel F. Scalabrini Machado, Angelo H. Lira Machado, Heibbe C. B. de Oliveira

**Affiliations:** † Laboratório de Estrutura Eletrônica e Dinâmica Molecular, 67824 Universidade Federal de Goiás, Goiânia, GO 74690-900, Brasil; ‡ Laboratório de Modelagem de Sistemas Complexos, Institute of Chemistry, 28127 Universidade de Brasília, Brasília, DF 70910-900, Brasil; § Laboratório de Isolamento e Transformação de Moléculas Orgânicas, Institute of Chemistry, Universidade de Brasília, Brasília, DF 70910-900, Brasil

## Abstract

Herein, we present a comprehensive computational study of the Hurd–Claisen rearrangement in both ester- and nitrile-containing substrates under oriented and nonoriented external electric fields (EEFs). Using high-level DFT calculations, we systematically mapped how EEFs perturb dipole–field interactions and transition-state geometries across a wide range of aryl and alkyl substituents. Fields aligned along the traditional reaction axis impart only modest differential stabilization (0.5–1.0 kcal·mol^–1^), insufficient to reverse *E*/*Z* ratios except in nearly degenerate cases, whereas molecular-axis alignment boosts this difference to over 2 kcal·mol^–1^, reliably inverting selectivity by maximizing dipole overlap. To bridge theory and experiment, we have applied the gold–thiolate anchoring in our model, harnessing substrate polarization to amplify transition-state stabilization from roughly 5 kcal·mol^–1^ in the free system to over 20 kcal·mol^–1^ at higher field strengths. We further show, via isotropic field ensembles weighted by Boltzmann and von Mises–Fisher statistics, that even nonoriented fields can impose a stereochemical bias of at most 2.5 kcal·mol^–1^ through dipole torque. These findings establish dipole orientation as a quantitative predictor of EEF-mediated stereocontrol and provide concrete blueprints for deploying “invisible catalysts” in flow reactors, surface-confined assemblies, and microreactor platforms for reagent-free, on-demand synthesis of stereochemically defined molecules.

## Graphical abstract

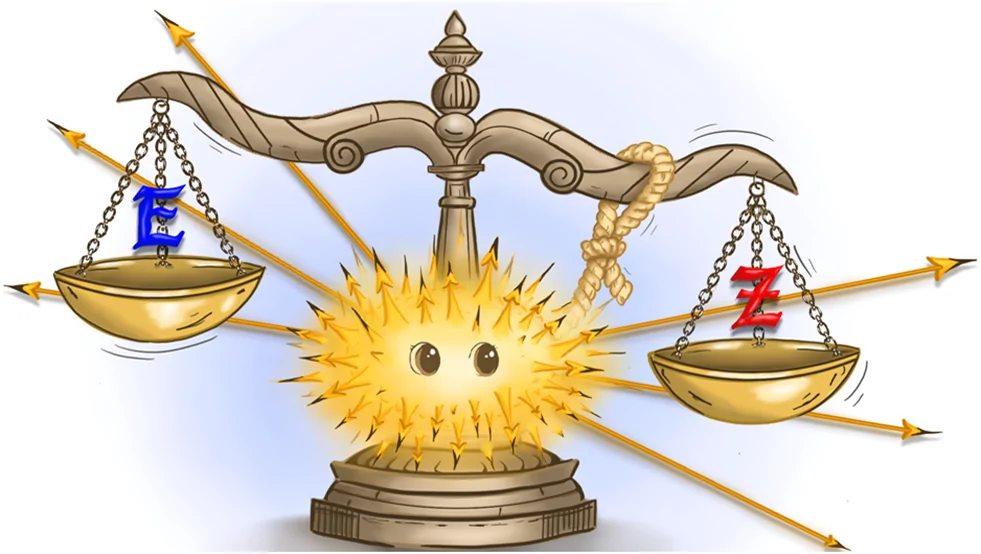

## Introduction

The Claisen rearrangement has long served as a cornerstone reaction for forging new C–C bonds via a concerted [3,3]-sigmatropic shift.^1–4^ Over the past century, it has found widespread application in the synthesis of natural products,^5,6^ pharmaceuticals,^7,8^ and functional materials.^9–11^ The Hurd–Claisen variant, which converts allylic alcohols into allyl enol ethers, enables access to synthetically challenging γ,δ-unsaturated carbonyl frameworks.^3,12^ Traditionally, stereocontrol in these rearrangements has been rationalized by Faulkner and Petersen’s steric model, in which repulsion between bulky substituents in the axial transition state directs product formation toward the equatorial isomer.^13^

An early study reported unprecedented E-stereoselectivity in the ester-containing Hurd–Claisen rearrangement of aryl-derived Morita–Baylis–Hillman adducts,^12^ highlighting electrostatic interactions as a non-negligible factor in the isomerization process. Building on this, our previous density functional theory (DFT) investigations^14^ expanded the mechanistic framework by applying Shubin’s energy decomposition analysis,^15^ revealing that variations in dipole moments across the central transition-state structure are the primary driver of selectivity between the equatorial and axial pathways. Despite these insights into intramolecular charge–dipole effects, a dynamic and externally tunable approach to stereocontrol in Claisen rearrangements remains unexplored.

In recent years, both theoretical and experimental studies have demonstrated that external electric fields (EEFs) can profoundly influence reaction pathways. Mechanistic DFT investigations have shown that aligning an applied field with evolving molecular dipoles can stabilize specific transition states, with field-induced geometric distortions playing a secondary role due to the high field strengths required to significantly alter geometry.^16–20^ Molecular dynamics simulations further reveal solvent screening effects that attenuate the field’s impact on the reactive core.^21^ Experimentally, Gorin and co-workers provided the first demonstration of field-driven stereocontrol, using an insulating polymer film between charged plates and an immobilized rhenium–porphyrin catalyst to achieve complete diastereoselectivity in an intramolecular rearrangement.^22–25^ However, it was the landmark single-molecule STM study by Aragonés et al. in 2016, showing a 5-fold rate enhancement in a Diels–Alder reaction under a precisely aligned electric field, that catalyzed widespread interest in oriented EEFs (OEEFs). Since then, numerous high-impact studies have explored electric field-mediated catalysis, transforming a theoretical concept into a powerful tool for manipulating antibiotic degradation in wastewater,^26,27^ reaction stereoselectivity,^16,22,23,28^ electron transfer,^29^ and catalysis.^30–33^

The ability to invert diastereomeric ratios through simple external controls has revolutionized the on-demand synthesis of stereochemically defined compounds,^23,34^ with direct implications for pharmaceutical development and functional polymer design. By leveraging OEEFs as “invisible catalysts”, providing catalytic acceleration and selectivity without consuming chemical reagents,^17,35–37^ and integrating field-responsive stereocontrol into continuous-flow platforms,^24^ it becomes possible to digitally program reaction outcomes without altering reagents, leaving no chemical footprint, and enabling truly reagent-free catalysis.

Herein, we report a comprehensive computational investigation of stereoselective Claisen rearrangements under EEFs. We quantify dipole–field interactions in both *E* and *Z* transition states and assess their impact on thermally corrected Gibbs free energies within the Curtin–Hammett framework.^14,38^ Four complementary field strategies are explored: (i) fields aligned along the traditional reaction axis;^36,39^ (ii) fields aligned with the dipole-focused molecular axis; (iii) substrate anchoring to gold–thiolate surfaces to enforce oriented fields and amplify polarization effects;^35,40^ and (iv) isotropic field ensembles to capture the influence of nonoriented environments.^17^ Together, these approaches provide mechanistic insights and practical design rules for leveraging oriented and nonoriented fields as noninvasive “invisible catalysts” in flow, surface-confined, and microreactor platforms.

## Methods

To extend our previous study of the Hurd–Claisen rearrangement to include the influence of EEFs, we examined both the ester-containing (Hurd-Est) and nitrile-containing (Hurd-Nit) variants. A series of organic substituents, *para*-aminophenyl (PhNH_2_), *para*-nitrobenzene (PhNO_2_), phenyl (Ph), methyl (Me), ethyl (Et), isopropyl (SPr), isobutyl (*i*-Bu), and tert-butyl (*t*-Bu), were evaluated (Figure 1A). The initial transition-state geometries were taken from our previous study,^14^ in which intrinsic reaction coordinate (IRC) calculations had already been performed to confirm the proper connectivity of each transition state to the corresponding reactant and product minima. These structures were then fully reoptimized at the M06–2*X*/6–311+G­(d,p) level, with single-point energy corrections at M06–2X/def2-TZVPP. Frequency calculations confirmed the transition-state character by identifying a single imaginary mode corresponding to the [3,3]-sigmatropic shift. Thermochemical corrections were obtained at 373 K (to maintain comparability with experimental results) using Grimme’s quasi-harmonic approximation (100 cm^–1^ cutoff), implemented in GoodVibes.^41^ According to the Curtin–Hammett principle, the predicted E/Z selectivity depends exclusively on the relative Gibbs free energies of the competing transition states, and therefore, reactant and product energies do not influence the stereoselectivity analysis. All electronic structure calculations were carried out with Gaussian 16.^42^

**Figure 1:**
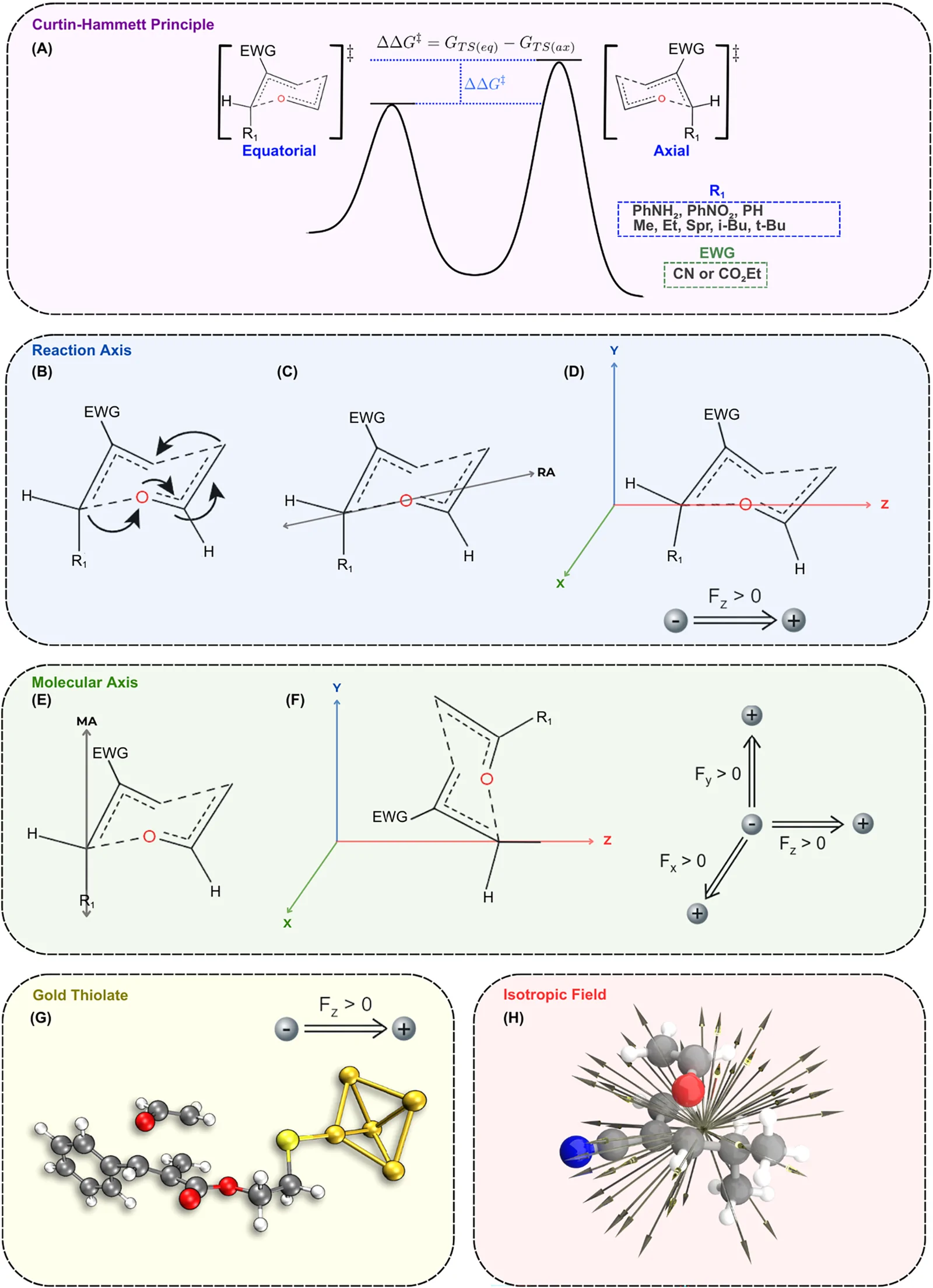
Equilibrium between conformational isomers of Hurd–Claisen TS’s under the Curtin–Hammett principle (A). Representation of the alignments used in this work. The reaction axis is first inferred from the electron flow, as shown in (B). Then, it is explicitly defined in (C) and aligned with the *Z*-axis of the Cartesian coordinate system in (D), where an EEF is applied. Similarly, the molecular axis is determined as the vector connecting the two most distant atoms in the molecule (E). This axis is then aligned with the *Z*-axis, and an electric field is applied along the *X*, *Y*, and *Z* components (F). A gold–thiolate system is proposed as an model in (G). The isotropic field is represented in (H), where random fields of equal magnitude are applied to different positions in the molecule.

Due to the interdependent design of the study, specific methodological choices such as whether field effects were introduced via single-point correction or full optimizations are discussed in the corresponding Results and Discussion subsections. We assessed a range of OEEFs varying from −8.00 × 10^–3^ to 8.00 × 10^–3^ au (≈ ±0.41 V/Å). This range was selected because it falls within the electric field strengths typically employed in STM-tip OEEF experiments (0.3–1.0 V/Å).^36^

We quantified the influence of OEEFs on stereoselectivity within the Curtin–Hammett framework^38^ by computing

$$\Delta \Delta G^{‡}\left(F\right)=G_{Z}^{‡}\left(F\right)-G_{E}^{‡}\left(F\right)$$

where *G*_*E/Z*_^‡^(*F*) represents the Gibbs free energies of the *E* and *Z* transition states under a given OEEF. Variations in *Δ*Δ*G* ^‡^(*F*) translate into altered diastereomeric ratios via the Boltzmann distribution, thus providing a direct quantitative link between the electric field and stereoselectivity.

OEEFs were applied along two principal directions as proof-of-concept tests to evaluate their impact on both stereoselectivity and overall reactivity. The first orientation was the reaction axis, inferred from curly arrow representations of electron flow^31,36,39^ and aligned with the Cartesian *Z*-axis (Figure 1B–D). The second was the molecular-axis, defined as the vector connecting the two most distant atoms in the transition-state structure, also aligned with the *Z*-axis (Figure 1(E,F)). To automate molecular alignment and atom reordering along these axes, we developed the Python package MoleKing, available via https://pypi.org/project/MoleKing/PyPI (pip install MoleKing).

Given the limitations of strictly oriented external electric field (OEEF) experimental setups, particularly the challenge of achieving precise alignment between the field and molecular axis, we built on our proof-of-concept tests for reaction- and molecular-axis control by simulating two experimentally feasible alternatives. First, we modeled substrate anchoring to a gold–thiolate cluster (Au_5_) to enforce a fixed field orientation and enhance polarization effects (Figure 1G). Second, we employed an isotropic field approach, applying equal-magnitude electric field vectors in randomized orientations around the transition-state geometry, to assess stereocontrol under truly nonoriented electric fields (Figure 1H).

## Results and Discussion

We begin our discussion on the application of OEEFs along the reaction axis. Later, we will shift attention to the molecular axis. These approaches provide complementary insights into how OEEFs modulate the stereochemical landscape of Hurd–Claisen rearrangements.

### Reaction-Axis Rule

Following the recommendations of Shaik et al.,^16,36,39^ the EEF was aligned with the reaction axis, defined by the electron flow direction associated with bond formation and bond-breaking events, and the geometry was fully optimized (Figure 1B–D). The relative electronic energies as a function of the applied field strength (−8.00 × 10^–3^ to 8.00 × 10^–3^ au) for the Me-substituted TSs are summarized in Figure 2A. We observed that both positive and negative field orientations stabilize the TSs; notably, the *E*-isomer benefits more under +*F*_*z*_, reaching up to 3.44 kcal·mol^–1^ stabilization. Under – *F*_*z*_ orientation, the stabilization drops to 2.80 kcal·mol^–1^, reflecting a 23% reduction favoring the positive orientation. This stabilization aligns with the trend reported by Wang et al. for the Mn-corrolazine-catalyzed CH_4_ oxidation reaction,^31^ where the OEEF substantially reduces the energy of the activated complex compared to the field-free case in both directions.

**Figure 2:**
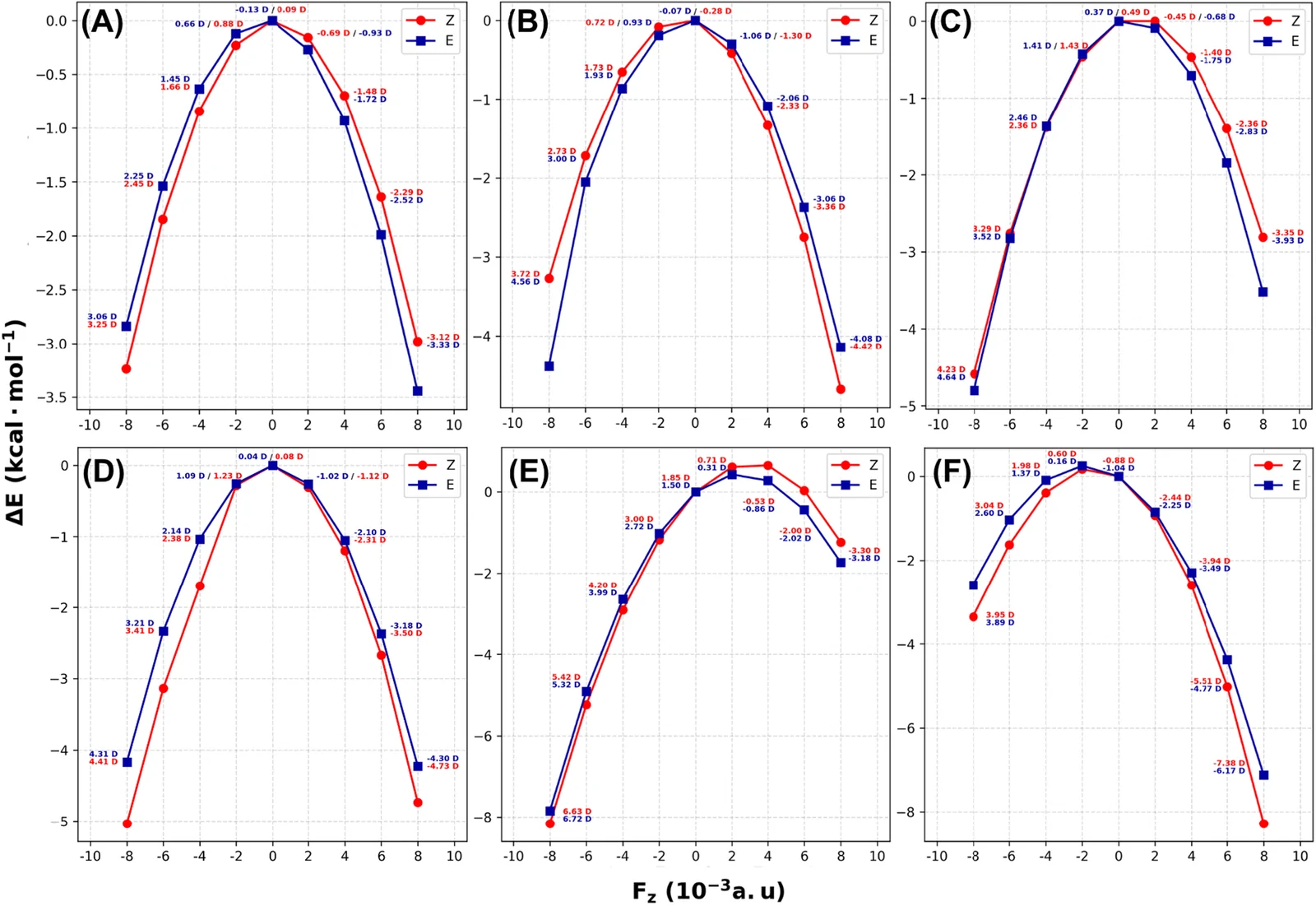
Relative electronic energy differences (with respect to *F*_*z*_ = 0) of transition states for the Hurd-Est rearrangement of (A) CH_3_, (B) IsoBut, (C) *t*-But, (D) Ph, (E) NO_2_Ph, and (F) NH_2_Ph substituents. The energy difference was computed as Δ*E* = *E*(*F*_*z*_)–*E*(*F*_*z*_ = 0).

This asymmetric behavior extends across different substituents, as shown in Figure S1 for Et and SPr groups. Interestingly, the bulky IsoBut substituent (Figure 2B) inverts the preference, favoring the *Z*-isomer under +*F*_*z*_ and the *E*-isomer under – *F*_*z*_ ones. For the other bulky t-But substituent, the *Z*-isomer was more stable under both field orientations (Figure 2C). This trend underscores the subtle interplay between the electronic and steric effects in modulating the dipole–field interactions. Despite these deviations, overall asymmetrical stabilization persisted in both field directions. As expected, the *ẑ*-component of the dipole moment followed the direction of the OEEF. For the *Z*-isomer, the *ẑ*-dipole moment ranges from −3.12 to 3.25 D, while in the axial *E*-isomer, it ranges from −3.33 to 3.06 D, for the Me-substituted TS. To probe this further, we analyzed the aryl substituents (chosen for their enhanced dipole moments and polarizability, Figure 2D–F), to compare them with the unprecedented stereoselectivity reported by Silva and co-workers for the alkyl substituents.^12^ Their stronger dipole moments predictably amplify OEEF interactions, leading to greater control over *E*/*Z* proportions. For the Ph-substituted TS, the *Z*-isomer was more stable in both field directions (Figure 2D). With the inclusion of an electron-withdrawing group (EWG) and electron donor group (EDG) in the aryl moiety, NO_2_Ph and NH_2_Ph, respectively, the former is first destabilized at the initial part of the OEEF with *F*_*z*_ > 0, whereas for the latter, the same occurs with *F*_*Z*_ < 0. However, as *F*_*z*_ becomes more positive for NO_2_PH (more negative for NH_2_PH), the *ẑ*-component flips its direction, and the field–dipole interactions stabilize the TSs again. A comparable effect was reported by Wang et al.^31^ The impact of OEEFs on geometric parameters was assessed by computing the root mean square deviation (RMSD) with respect to the field-free geometries, but no significant changes were observed (Figure S4). Similar findings were reported by Liu and Liu.^19^

The *Z*/*E* ratio can be estimated from the difference in the Gibbs free energy between the transition states of both isomers, *Δ*Δ*G* ^‡^. A negative value of this difference indicates a more stable *Z*-isomer at the TS. Typically, the orientation of the OEEF is expected to induce stabilization (catalysis) or destabilization upon field inversion (inhibition).^16,36^ However, in the Hurd–Claisen rearrangement, the symmetric and parallel alignment of forming C–C and breaking C–O bonds results in comparable stabilization effects for both positive and negative OEEF orientations along the reaction axis. The Gibbs free energy differences between Hurd-Est transition states are shown in Figure 3A.

**Figure 3:**
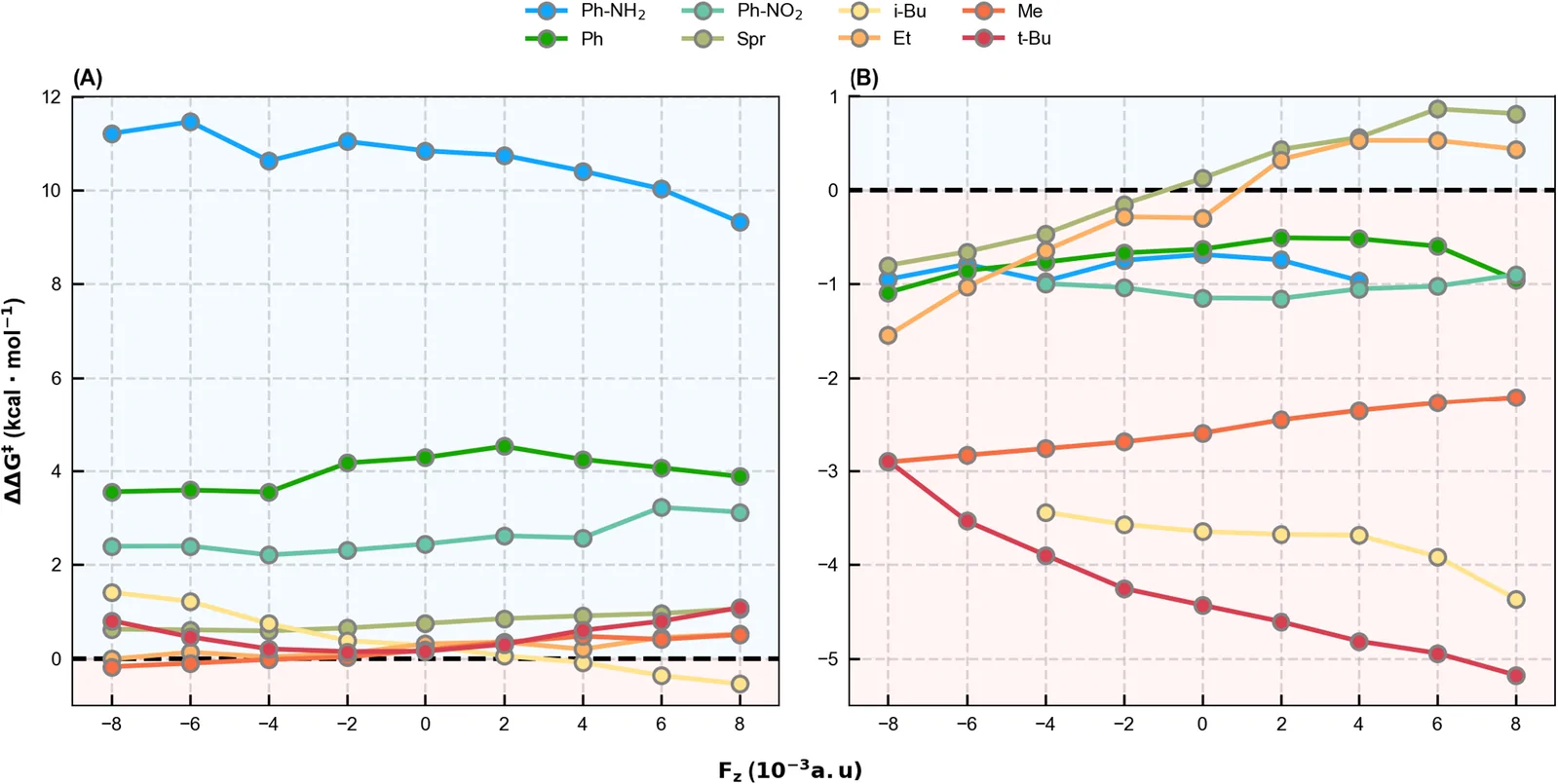
Gibbs free energy differences of transition states ΔΔ*G* ^‡^ for (A) Hurd-Est and (B) Hurd-Nit rearrangements. ΔΔ*G* ^‡^ was calculated using Grimme’s *quasi*-harmonic approximation at 373 K with the M06–2X/def2-TZVPP level of theory. The red-shaded region indicates where the *Z* isomer is favored over the E isomer.

The aryl substituents exhibit a strong preference for the *E*-isomer, with stereoselectivity remaining invariant to the OEEF strength or orientation (Figure 3A). This behavior arises from the asymmetric transition-state stabilization governed by the *ẑ*-component of the dipole moment.

Reaction-axis calculations revealed that the OEEF-driven stabilization depends on the dipole moment component aligned with the applied field (here, the *Z*-axis) computed in the absence of the field. For a small dipole moment (±0.1 D), stabilization is symmetric, as observed in Figure 2D, as minimal resistance to dipole inversion allows favorable (antiparallel) field alignment. In contrast, larger dipoles (±1 D) exhibit a more complex response, with initial destabilization at weak field strengths due to unfavorable dipole reorientation, followed by stabilization as the field overcomes this barrier. For example, in NO_2_Ph (Figure 2E), a positive OEFF initially destabilizes the transition state but stabilizes it at higher field strengths, while NH_2_Ph (Figure 2F) shows analogous behavior for −*F*_*z*_. This is attributed to the inherent alignment of the dipole, where a negative (positive) dipole stabilizes under +*F*_*z*_ (−*F*_*z*_) once the field exceeds the dipole reorientation threshold. This trend persists across all rearrangements, including the Hurd-Nit case (Figures S2 and S3).

Still focusing on Figure 3, our calculations reveal that the electric field fails to invert stereoselectivity for the cases where the barrier height differences are greater than Δ*G* ^‡^ ≈ ±1 kcal·mol^–1^. In systems with ΔΔ*G* ^‡^ ≈ 0, the stereoselectivity can be readily reverse and will be termed here as “indecisive” reactions. This result highlights the influence of substituent-dependent energy landscapes on the feasibility of OEEF control over reaction outcomes.

Comparing both rearrangements (Figures 2 and S2 with Figure 3), with the exception of the *tert*-butyl system, all reactions maintained the same trends of stabilization as a function of the OEEF. This highlights the idea that the EWG generally modifies only the intensity of the intramolecular electrostatic interactions and not their fundamental nature. Consequently, the OEEF remains capable of controlling the stereoselectivity of reactions with low barriers.

### Molecular-Axis Rule

The molecular axis is defined as the vector connecting the two most distant atoms in the molecule (Figure 1D). Similar to the reaction axis, this vector is aligned with the Cartesian *Z*-axis. However, in this case, the electric field is applied along all three Cartesian directions at ± 1.00 × 10^–4^ and ± 6.00 × 10^–4^ au (Figure 1E). The use of the molecular axis results in an alignment not dependent on the electron flow, more suitable for achieving stereoselectivity control.^17,36^ As observed for the reaction-axis method, geometric displacements induced by field perturbation are negligible (Figure S4). Thus, full TS optimizations under the field were omitted; instead, field perturbations were evaluated by single-point calculations on the field-free TS geometry.

Figure 4 summarizes the energy profiles for the Hurd-Est (A) and Hurd-Nit (B) rearrangements under different OEEF orientations. A striking difference is observed between the two systems: while the Hurd-Est rearrangements consistently favor the *E*-isomer, the Hurd-Nit reactions exhibit a clear preference for the *Z*-isomer. Another contrasting trend lies in the readily invertible nature of the Hurd-Nit variant, in which stereoselectivity can be reversed with a relative ease, whereas its ester-based counterpart rarely exhibits such inversion. As a general trend, the magnitude of the OEEF primarily modulates the extent of stabilization rather than altering its directional preference. For instance, if a field of 6.00 × 10^–4^ au stabilizes the *Z*-isomer, a weaker field (e.g., 1.10 × 10^–4^ au) will also favor the same isomer, albeit with reduced efficacy. This trend persists upon field reversal, although the favored isomer may switch (e.g., from *Z* to *E*). These divergences arise from distinct electrostatic interactions: the nitrile electron-withdrawing group stabilizes the *Z*-isomer, whereas the ester electron-withdrawing group promotes *E*-isomer stabilization. These opposing effects underscore how subtle variations in electrostatic interactions can decisively alter OEEF-driven reaction pathways.

**Figure 4:**
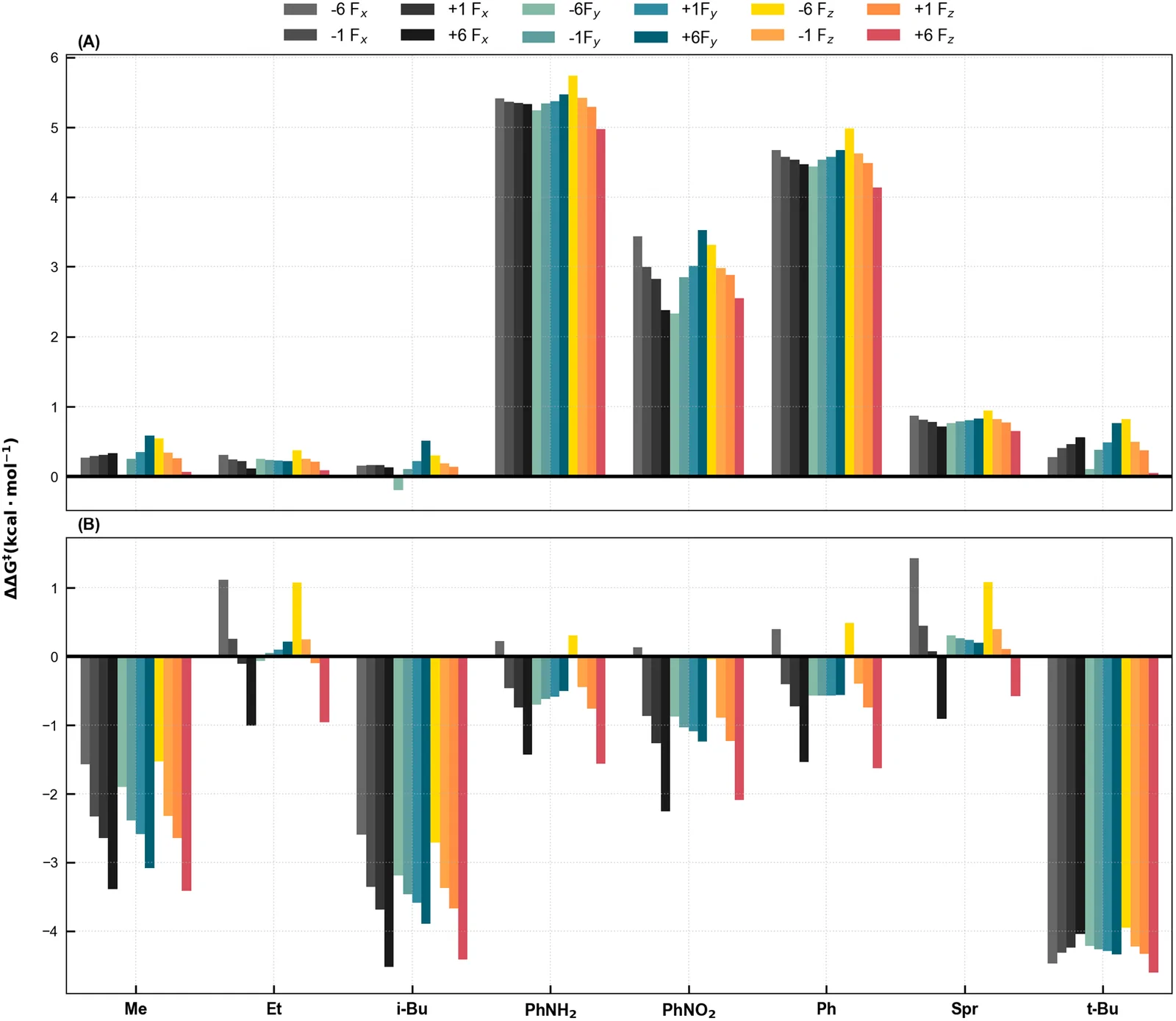
OEEF effect in the Me, Et, SPr, *i*-Bu, *t-*But, Ph, PhNO_2_, and PhNH_2_ for (A) ester-containing and (B) nitrile-containing Hurd–Claisen rearrangements. The OEEF was applied along all three axes with two different magnitudes, 1.00 × 10^–4^ and 6.00 × 10^–4^ au, in opposite directions. These results were obtained with single-point calculations at the M06–2X/def2-TZVpp level of theory.

Critically, varying the OEEF magnitude alone rarely overrides the inherent stereoselectivity in reactions with a stronger isomer preference (for instance, Hurd-Est, where ΔΔ*G* ^‡^ > 0). However, for “indecisive” reactions such as the Me- and butyl-substituted Hurd-Est rearrangements (where competing transition states are nearly degenerate, i.e., small ΔΔ*G* ^‡^), the OEEF can effectively control the stereoselectivity. Hence, our calculations demonstrate the utility of OEEFs as a selective tool for reactions with small differences in ΔΔ*G* ^‡^. The same observations extend to the Hurd-Nit reactions. Here, aryl substituents (Ph, NO_2_Ph, and NH_2_Ph) exhibit tunable stereoselectivity owing to their “indecisive” energy barriers (ΔΔ*G* ^‡^ ≈ 0), enabling precise control via OEEF regardless of their orientation with respect to the molecular axis. Recall that when we used the reaction-axis approach, the OEEF failed to tune the stereoselectivity across all substituents (with the exception of the Et substituent).

The molecular-axis orientation had a superior performance in altering the reaction stereoselectivity compared to the reaction-axis approach, even at lower field strengths. Our calculations show that closely related *Z*/*E* transition states exhibit stereoselectivity inversion dependent on the OEEF magnitude. For the Hurd-Est rearrangement, a negative *y*-oriented field inverted the selectivity in *IsoBut*-substituted reactions. In the Hurd-Nit case, −*F*_*x*_- or −*F*_*z*_-oriented fields induced inversion for Et, SPr, and all aryl substituents, whereas any +*F*_*y*_-oriented field inverted Et and SPr reactions. As stated by Shaik, off-reaction axis OEEFs preferentially control selectivity rather than reaction rates, as they avoid direct modification of reactive bonds.^39^

### Anchored Transition States by Thiolate Linkers on Gold Substrates

Despite the advantages of molecular-axis alignment for stereocontrol (especially in reactions with near-degenerate ΔΔ*G* ^‡^ free energies), the approach faces significant experimental challenges, chiefly due to the difficulty in aligning an external field along an abstract molecular coordinate. Prior studies have demonstrated that immobilization of substrates on metallic surfaces can enforce fixed orientations relative to the applied field,^23,35,40,43^ thus enhancing the catalytic performance. Notably, Shaik and collaborators used aromatic anchoring in the Menshutkin and Diels–Alder reactions to align the reactive axis with the field direction,^40,43^ while Coote’s experimental design employed dienes tethered to gold to achieve a similar alignment in Diels–Alder catalysis.^35^ These approaches prioritized reaction-axis alignment to maximize field-induced reactivity.

Here, only the Au_5_–thiolate anchoring moiety was kept fixed during the geometry optimizations, while the reactive organic framework remained fully relaxed. Furthermore, based on our previous analysis of field-induced structural perturbations (Reaction-Axis Rule), where optimized transition states under the OEEF showed negligible geometric deviations relative to the field-free structures (Figure S4), all field-dependent calculations for the tethered model were performed as single-point energy evaluations on the zero-field optimized geometries. Consequently, the restrained optimization protocol still allows local conformational adaptation and short-range noncovalent interactions between the flexible organic fragment and the anchored Au-thiolate region, while avoiding artificial distortions of the finite Au_5_ cluster under strong electric fields.

Motivated by our findings that molecular axis-aligned fields can exert stronger control over stereoselectivity than reaction-axis alignment, we sought to adapt a similar anchoring strategy, but this time, we tailored to mimic the molecular-axis model, as shown in Figure 1G. We explore how anchoring through ester-functionalized thiols (commercially available and synthetically accessible) can replicate the effects of molecular-axis alignment while offering practical advantages over prior aromatic or diene-based anchoring methods. Importantly, this methodology is inapplicable to the Hurd-Nit rearrangement due to the absence of anchoring-compatible functional groups and is thus restricted to the Hurd-Est systems.

The Hurd-Est Me- and Ph-substituted systems were covalently immobilized onto a gold–thiolate Au_5_ cluster via the ester electron-withdrawing group, as illustrated in Figure 5. This anchoring strategy, inspired by previous gold-surface models^40^ enables directional control of the applied electric field. A uniform OEEF ranging from −8.00 × 10^–3^ to 8.00 × 10^–3^ au was applied along the Cartesian *Z*-axis, perpendicular to the gold surface, to simulate feasible experimental configurations. The structural model, including geometry and electronic parameters of the Au_5_ cluster, was adopted from Ramanan et al.^40^ To isolate the field’s influence on the reactive framework, the gold–thiolate fragment was held fixed during all geometry optimizations, while the remainder of the molecular system was fully relaxed at the M06–2X hybrid exchange-correlation functional with the 6–311+G­(d,p) basis set for light elements and SDD pseudopotential for Au atoms. Following geometry convergence, single-point energy calculations were performed to evaluate the field perturbations. Figure 5A,B illustrates the progressive stabilization of the optimized transition states of the Hurd-Est CH_3_ and Ph systems under increasing OEEF magnitudes.

**Figure 5:**
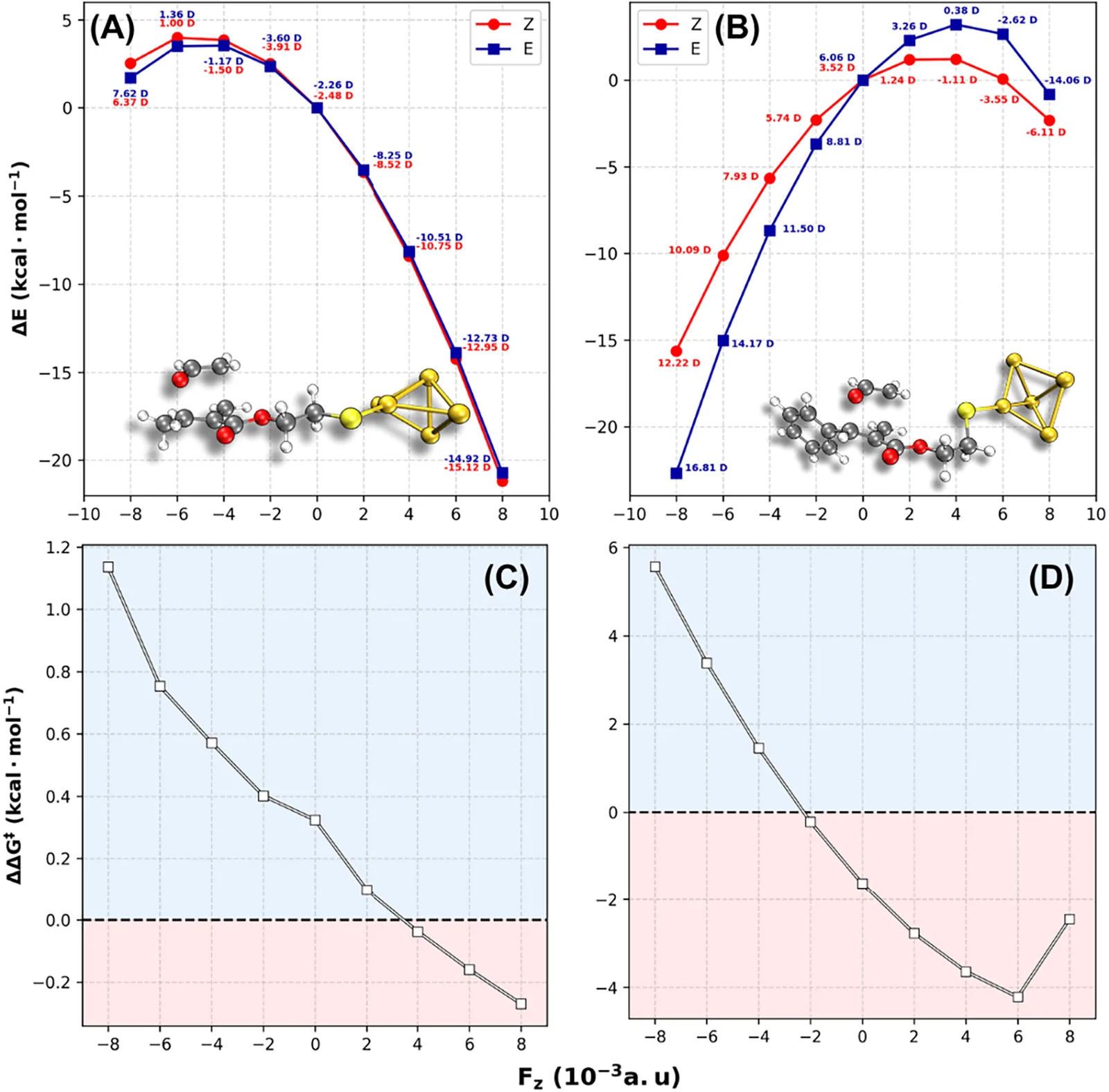
Field-induced effects in gold-surface immobilized Hurd-Est rearrangements. (A, B) Transition-state energy stabilization was computed as Δ*E* = *E*(*F*_*z*_) – *E*(*F*_*z*_ = 0) and (C, D) Gibbs free energy differences (ΔΔ*G* ^‡^) for the Me (left)- and Ph-substituted (right) systems. Calculations employed Grimme’s *quasi*-harmonic approximation at 373 K (M06–2X/def2-TZVPP//M06–2*X*/6–311+G­(d,p)). Red regions (C, D) indicate *Z*-isomer preference (ΔΔ*G* ^‡^ < 0). Gold substrates are coupled via thiol linkages to enforce directional field effects.

The surface-anchored systems exhibit notably enhanced stabilization compared with previous cases, attributable to the gold substrate’s high polarizability. This property amplifies OEEF interactions and induces substantial dipole modulation through substrate polarization, consistent with the findings of Ramanan et al.^40^

A key contrast emerges between the CH_3_- and Ph-anchored systems with their stabilization trends inverting due to opposing dipole characteristics. While the Ph transition state displayed a positive *ẑ*-component of the dipole, the CH_3_ system exhibits a negative one. Consequently, each structure is preferentially stabilized by a field oriented opposite to its dipole moment and initially destabilized by a field in the same direction. These results demonstrate how metal substrates can actively modulate the molecular response to electric fields where dipole–field interactions ultimately lead to net stabilization in both systems as the dipole becomes antialigned with the applied field.

The ΔΔ*G* ^‡^ trends in Figure 5C,D reveal significant substrate-mediated stereocontrol. For the Ph system, field-free gold anchoring alone reverses the previously reported *E*-selectivity, establishing *Z* preference despite their weaker field-induced modulation. In contrast, the CH_3_ system remains largely unaffected, retaining its preference for the *E* isomer in field-free case. At extreme field strengths (8.00 × 10^–3^ au), we observe reduced stereodifferentiation and re-emerging *E*-preference. However, this behavior is unlikely to impact experimental implementations, as practical field strengths typically remain below this threshold by an order of magnitude. However, to investigate this apparent anomaly, we analyzed the wave function stability of the corresponding transition states (*E*- and *Z*-isomer) and identified the emergence of an open-shell singlet configuration only for the *E*-isomer, as revealed by a broken-symmetry solution. With weaker field strengths, no broken-symmetry solution was observed. This behavior resembles the findings reported by Ramanan et al.^40^

The influence of the gold–thiolate linkers on the properties of the Hurd–Claisen reaction was also evaluated by analyzing the Mulliken group charges of the molecular fragments defined in Figure S16, as well as the total molecular dipole moment in both field-free conditions and under applied ±*F*_*z*_ fields, as shown in Figure 6. This analysis is conceptually inspired by the work of Ramanan et al.,^40^ who emphasized the role of field-induced charge migration and dipole modulation in governing reactivity and selectivity under OEEFs.

**Figure 6:**
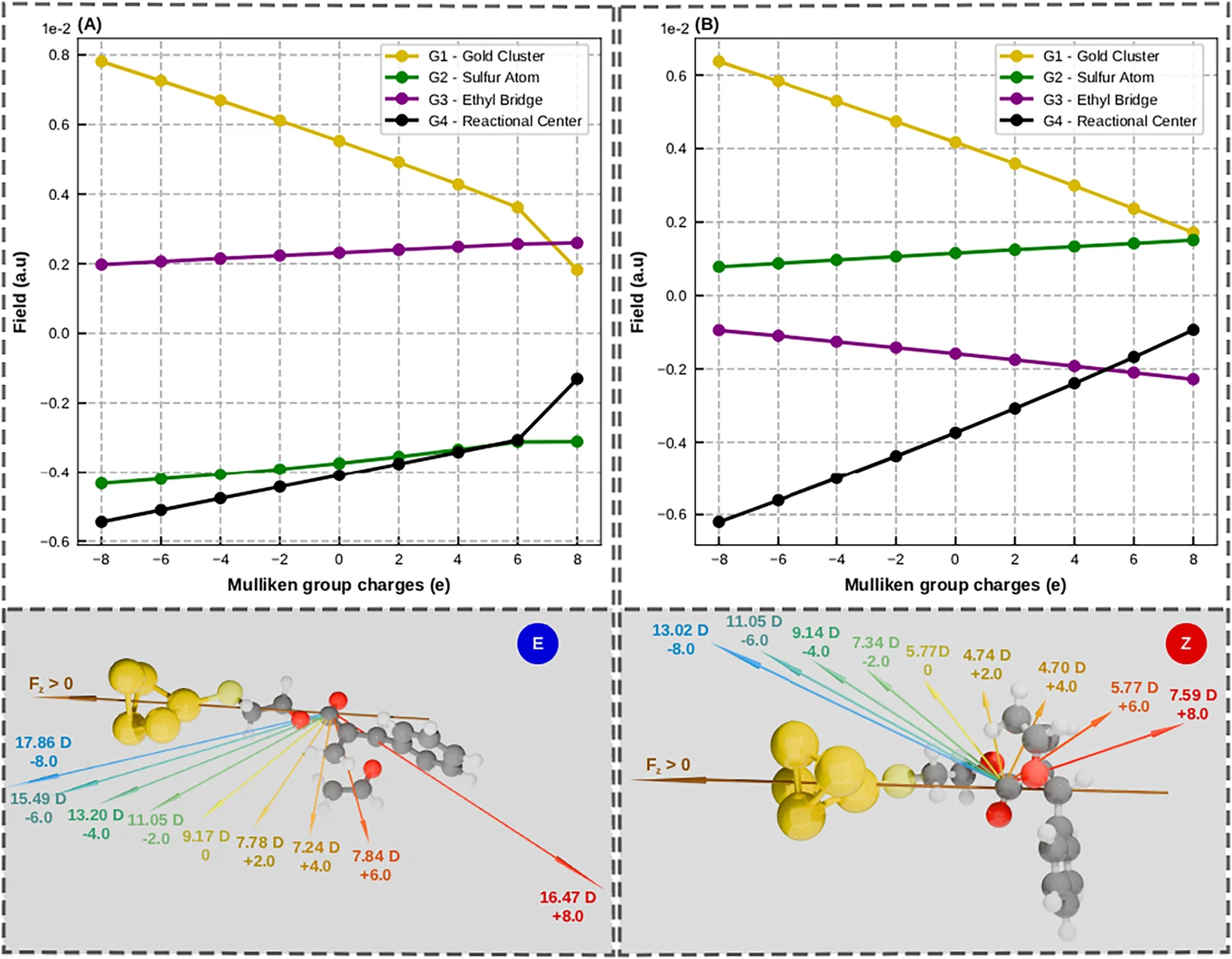
Mulliken group charges of the molecular fragments defined in Figure S16 as a function of the OEEF for (A) *E*- and (B) *Z*-isomer of the Ph-substituted Hurd–Claisen rearrangement. The lower panels show the positive direction of the external field (along the *z*-axis) and the corresponding total molecular dipole moments (in Debye) as a function of both strength and orientation of the field (in 10^–4^ au) for each isomer.

In the absence of an external field, there is a clear charge separation more pronounced between fragment G1 (gold cluster) and G2 (sulfur atom), but the separation extends from the positively charged G3 (ethyl bridge) to the negatively charged G4 fragment (the reaction center). We observed that this charge separation at *F*_*z*_ = 0 is more significant for the *E*-isomer than for the *Z*-isomer. Thus, the linkers introduce local dipoles whose electric fields are the underlying reason for the inversion for *Z*-preference observed at *F*_*z*_ = 0 in Figure 5D for Ph-substituted Hurd-Est rearrangement, showing also in the case of the reactions studied here that the linker moieties may not remain innocent when OEEFs are applied on the system.^40^ Also, we note from the lower panels of Figure 6 that the total molecular dipole moment at *F*_*z*_ = 0 is almost orthogonal to the external field in the *E*-isomer but that there is some positive projection in the *Z*-isomer. These relative dipole orientations effectively explain the initial destabilization of the transition state for *F*_*Z*_ > 0 observed in Figure 5B.

Upon application of a positive electric field (+*F*_*z*_), we observe a pronounced polarization effect characterized by charge density shifting away from the reaction center (G4) toward the Au cluster (G1). In contrast, the connecting groups (G2 and G3) exhibit only modest changes, indicating a limited participation in the polarization process. This trend is reflected in the shallow slopes of their charge versus field curves in Figure 6. Under +*F*_*z*_, the total dipole moment increases in magnitude and reorients in the direction of the field, but the magnitude of the total dipole decreases accordingly.

When the field is reversed (−*F*_*Z*_), the direction of charge transfer is also reversed: G1 donates electron density to the other fragments, particularly toward the reaction core. As a result, the total dipole moment projects in the negative *z*-direction and reaches a larger magnitude compared to the +*F*_*Z*_ case. This enhancement arises from the more extensive charge separation induced by the greater electron donation from the Au cluster. In all cases, the total dipole moment reorients to remain aligned with the direction of the applied EEF, reflecting the system’s intrinsic tendency to minimize electrostatic energy through dipole–field alignment.

While directional fields can effectively guide selectivity in cases involving near-degenerate transition states, the inclusion of gold surface anchoring introduces additional charge redistribution effects capable of influencing stereochemical outcomes even in cases with more pronounced energy differences.

### Isotropic Electric Field

A persistent experimental limitation remains: the need for precise alignment between the reactive framework and the applied field.^24,35,39^ This raises a critical question: *Can comparable levels of stereocontrol be achieved using isotropic electric fields without requiring strict orientation of the molecule relative to the field*?

To address this, we implemented an isotropic field model by applying 50 distinct OEEFs with randomized orientations and a fixed strength of 4.00 × 10^–3^ au, Figure 1H for the Me-, Et-, SPr-, Ph-, and PhNO_2_-substituted Hurd-Nit system and Me- and SPr-substituted Hurd-Est variant. By removing alignment constraints, this approach provides a more general framework for probing field effects on stereoselectivity under experimentally relevant isotropic conditions.

To capture the full extent of field-induced geometrical and energetic perturbations, each field orientation was treated independently, with full geometry optimizations and thermochemical corrections performed for the resulting transition states. This allowed us to evaluate how field–dipole interactions influence barrier heights in a rotationally averaged system.

Figure 7A shows the results for the electronic energy differences (Δ*E*), and Figure 7B,C shows the dipole moment components of each isomer, in the case of the *SPr* derivative of the Hurd-Nit rearrangement.

**Figure 7:**
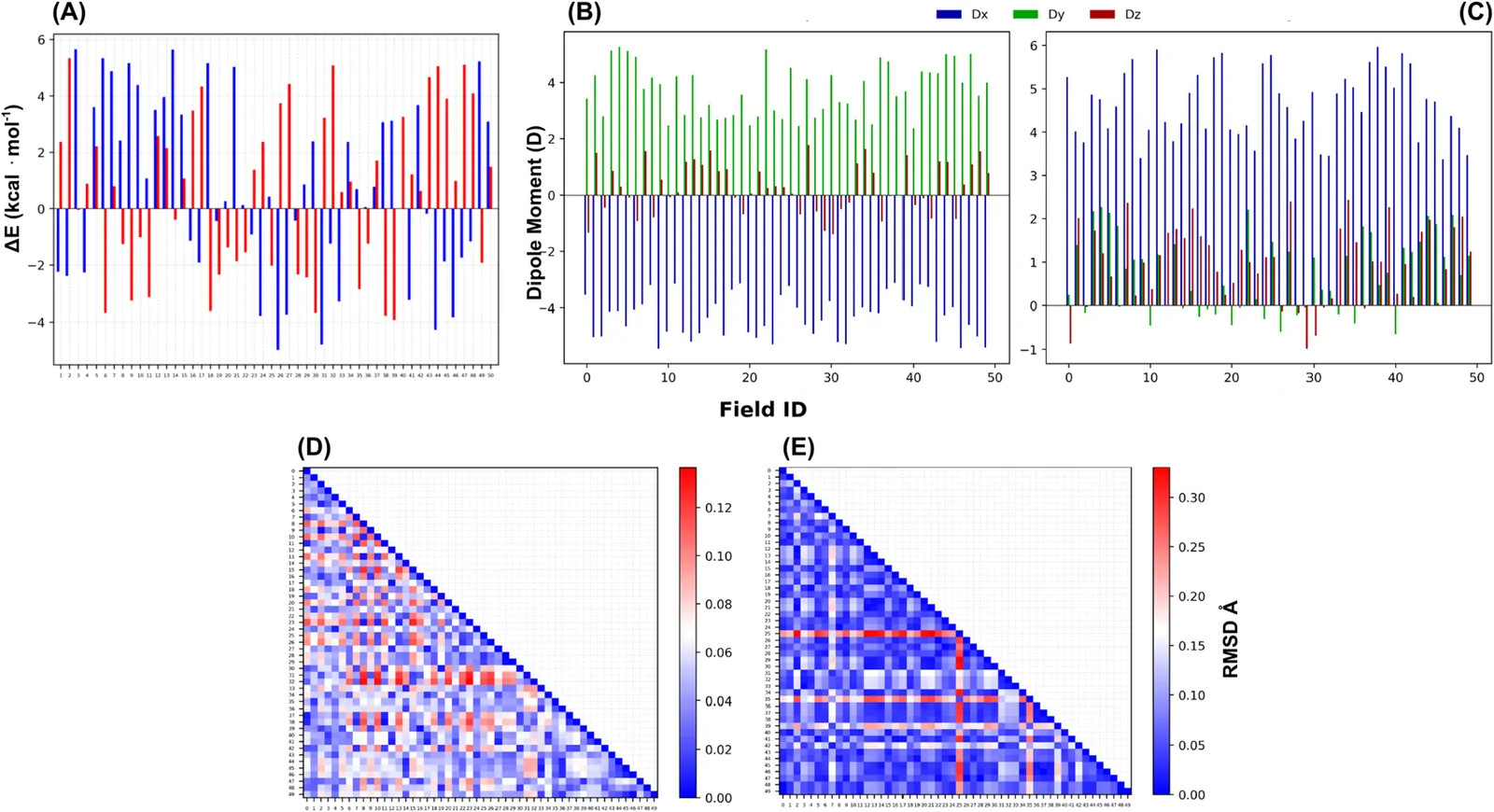
Field-induced perturbations in the SPr Hurd-Nit rearrangement under isotropic nonoriented electric field (4.00 × 10^–3^ au). (A) Transition-state energy changes for *E*- (blue) and *Z*-isomers (red) across 50 randomized field orientations (numbered 1–50). (B, C) Corresponding dipole moment components (*x*, *y*, *z*) for the *Z*- and *E*-isomers, respectively. (D, E) Root mean square deviation of the *E*- and *Z*-isomers, respectively. Note the different scales of the RMSD values for each isomer.

The distribution of electronic energy changes in Figure 7A demonstrates that the *E*- and *Z-*isomers respond differently to the same field ensemble, providing the basis for stereochemical discrimination. Consistent with previous findings, the EEFs alter the molecular dipole moment. This behavior persisted under isotropic field conditions, where the dipole moments exhibited significant orientation-dependent variability, as illustrated in Figure 7B,C. The dipole moments of both isomers are similar in magnitude but have opposite orientations for the *x̂*-component. The *E-*isomer displayed an overall negative dipole orientation, whereas the *Z-*isomer exhibited positive values for most of its components. This difference arises from the distinct component contributions. For the *E* isomer, there is a competition between the *x̂* and *ŷ* components, whereas the *Z* isomer is dominated by the *x̂* direction. This asymmetry implies that the electric field interacts with the dipole moment of each isomer differently. Consequently, *Y*-oriented OEEFs preferentially influence the *E* isomer, whereas *Z*-oriented fields along the reaction axis minimally differentiate between the isomers. To quantify geometrical changes, we performed RMSD analysis (Figure 7D,E), revealing that the *Z* isomer exhibits a slightly larger geometric flexibility at the transition state. Hence, the influence of the isotropic field on geometry does not contribute to stereocontrol. The remaining substituents, CH_3_, Et, Ph, and NO_2_Ph, in the Hurd-Nit rearrangements confirmed this trend (as shown in Figures S11 to S15).

These results contrast sharply with the reaction-axis OEEFs, where nonreactive field orientations induce more substantial dipole and energy changes. This trend is consistent with Shaik’s paradigm that off-axis fields offer superior stereocontrol by selectively modulating the transition-state electronic environments without requiring bond-axis alignment.^36,39^ As Shaik demonstrates, the need for precise geometric orientation is not a problem, since they will experience a torque from the OEEF, which naturally aligns molecular complexes.^39,44^

Owing to the complex relationship between the randomly oriented fields and stabilization energies (Figure 7A, for instance), which depends critically on dipole–field alignment, we quantified the average effect through arithmetic means of Gibbs free energies. For each isomer, we calculated the mean Gibbs free energy across all field orientations and then determined the average energy difference, ⟨ΔΔ*G* ^‡^⟩ = ⟨*G*_*Z*_^‡^⟩ – ⟨*G*_*E*_^‡^⟩, as shown in Table 1. Arithmetic averaging of the Gibbs free energy revealed multiple cases of stereoselectivity inversion compared to field-free conditions.

**Table 1:** Average Free Energy Differences, ⟨ΔΔ*G* ^‡^⟩, between *Z*- and *E-*Isomers for Hurd-Nit and Hurd-Est Rearrangements^a^

**reaction**	**substituent**	**arithmetic**	**Boltzmann**	**vMF**	**field-free** ^14^
Hurd-Nit	Me	–2.77	–2.14	–2.64	–2.64
	Et	0.48	0.98	1.10	–0.06
	SPr	0.27	0.67	0.53	0.25
	Ph	0.61	0.58	0.87	–0.25
	PhNO_2_	–0.64	–5.78	–3.51	–0.88
Hurd-Est	Me	–0.57	0.10	–0.46	0.13
	SPr	1.06	1.43	1.38	0.88

a Values (in kcal·mol^–1^) compare three averaging methods: arithmetic mean (Arithmetic), Boltzmann-weighted average (Boltzmann), and von Mises–Fisher distribution (vMF) as well as the field-free reference of Oliveira and co-workers.^14^

Experimentally, the applied electric field induced both structural relaxation and molecular reorientation, favoring antiparallel alignment. To account for this rotational freedom, we implemented an orientation-weighted averaging scheme based on dipole–field interaction energies, Δ*E* = 4.8*F⃗*·μ⃗,^17^ where perturbed dipole moments were used to compute Boltzmann-weighted (β_*i*_) Gibbs Free energy for each configuration, as follows

1 $$\beta_{i}=e^{-\left(\frac{{\mathrm{F⃗}}_{i}\cdot {\mathrm{\mu ⃗}}_{i}}{4.8k_{b}T}\right)}$$

In this weighting scheme, configurations with antiparallel dipole–field alignment receive higher statistical weight, whereas parallel alignments are effectively suppressed. The ensemble-averaged Gibbs free energy was calculated as follows

2 $$\left\langle G_{E\;\mathrm{or}\;Z}^{‡}\right\rangle =\frac{\sum_{i=1}^{n}\beta_{i}G_{i}}{\sum_{i=1}^{n}\beta_{i}}$$

where *G*_*i*_ is the Gibbs free energy under the *i*th sampled field. With this expression, we computed the average differences, ⟨ΔΔ*G* ^‡^⟩ = ⟨*G*_*Z*_^‡^⟩ – ⟨*G*_*E*_^‡^⟩, as presented in Table 1.

Boltzmann averaging reproduced the qualitative trends observed in the arithmetic means (including stereoselectivity inversions), albeit with an amplified magnitude owing to its exponential sensitivity to dipole–field interactions (e^–Δ*E*/*k*_B_ *T*^). As shown in Figure 8, this scheme disproportionately favors near-antiparallel alignments and effectively suppresses other configurations. However, Boltzmann weighting assumes instantaneous orientational equilibrium, which is a problematic premise for transition states, whose lifetimes (10^–13^ s)^45^ are typically several orders of magnitude shorter than the rotational diffusion time scales (≫10^–9^ s).^46^ Hence, the apparent CH_3_ inversion is likely an artifact of the overweighted antialigned states that are kinetically inaccessible. To resolve this bias while retaining the preferential alignment effects, we employed a von Mises–Fisher distribution scheme (Table 1). This approach better represents the partial orientational equilibrium achievable during the TS lifetime because it maintains the Boltzmann-like preference for antialignment and smoothly samples nonequilibrated configurations.

**Figure 8:**
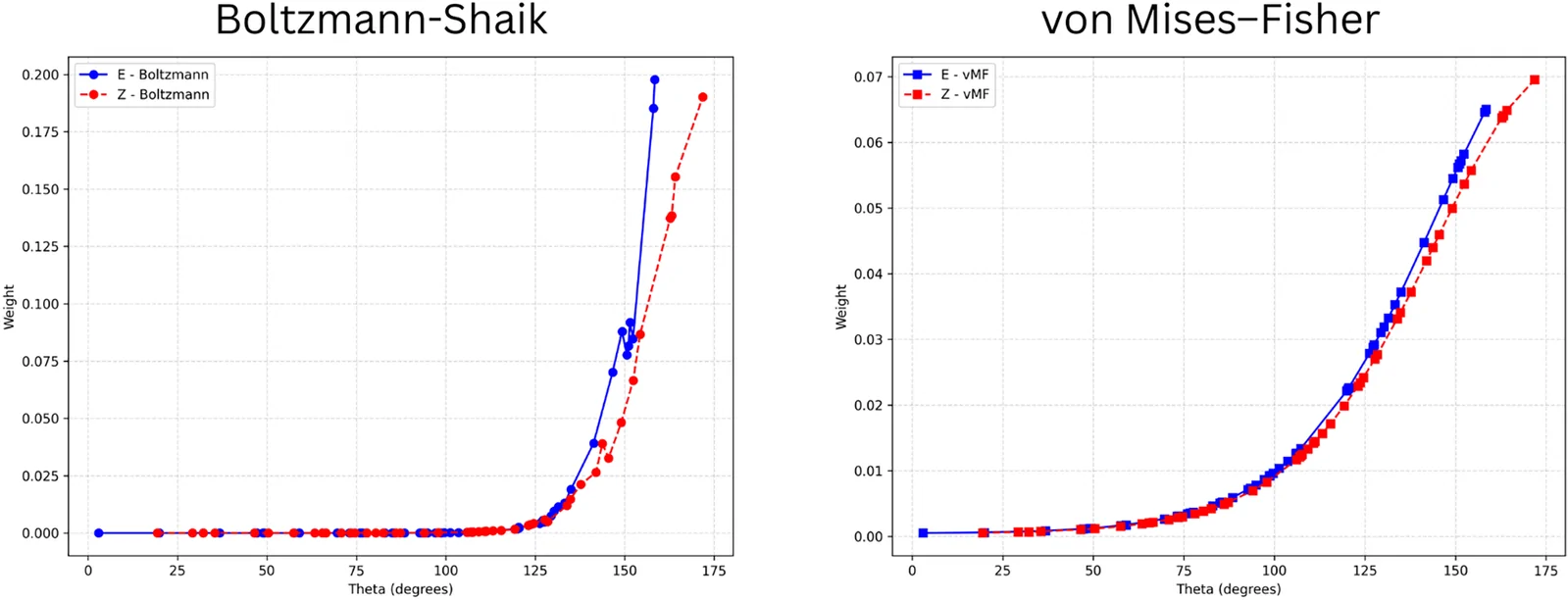
Statistical Weights of the SPr Hurd-Nit rearrangement under 50 randomly oriented electric fields (4.00 × 10^–3^ au), comparing Boltzmann (left) and von Mises–Fisher (right) distributions.

The von Mises–Fisher distribution^47,48^ is used for modeling directional data and is suitable for describing molecular dipole orientations. This distribution is governed by two key parameters: (1) a mean direction vector and (2) a concentration parameter κ that controls directional clustering. For the collection of EEFs, we defined the mean direction using the cosine of the angle between the dipole moment and the electric field vectors, with κ = 5 orientation specificity and sampling breadth. The resulting cosine-weighted distribution

3 $$f\left(\theta \right)=e^{\left(\frac{\kappa \left(1-\cos\theta \right)}{2}\right)}$$

preserves alignment preferences while minimizing biases from extreme orientations.

The Et and Ph Hurd-Nit rearrangements switch from *Z*-preference (field-free) to *E*-preference under an isotropic field. The SPr and NO_2_Ph rearrangements displayed a strengthened *Z*-preference. The CH_3_ Hurd-Est rearrangements inverted the selectivity from *E* to *Z*, while the Ph-substituted system strengthened its *E* preference. We also noted that the nitrile-containing Me-substituted system showed a minimal response.

Isotropic electric fields effectively affect stereoselectivity for reactions, although the magnitude and direction of the control depend critically on substituent properties. Previously, “decisive” reactions such as the Hurd-Est Ph case did not undergo a complete inversion of stereoselectivity, and the isotropic field was able to promote such inversions. Nevertheless, even in cases without stereoinversion, the relative isomer barrier can be manipulated. These findings establish isotropic electric fields as a versatile tool for stereocontrol.

## Conclusions

In conclusion, we have presented a comprehensive computational study on the application of OEEFs as noninvasive tools for controlling stereoselectivity in Hurd–Claisen rearrangements. By systematically comparing multiple substituents and field alignment strategies, we established that the molecular-axis approach is significantly more effective than the conventional reaction-axis method, achieving stereochemical inversion at field strengths up to 1 order of magnitude lower. This enhancement is rooted in the preferential alignment of the field with the molecular dipole rather than with reactive bonds, in line with Shaik’s theoretical paradigm for selectivity-focused catalysis. Furthermore, our implementation of a gold–thiolate anchoring model demonstrated that field-induced stabilization can be dramatically intensified through substrate polarization effects, with dipole–field interactions modulated by the intrinsic dipolar character of both the substituents and the metallic linker. Notably, the ability of these anchored systems to invert stereoselectivity even under moderate fields suggests experimental feasibility and opens avenues for electrostatic catalysis in real systems.

In parallel, the isotropic field model addressed a key practical challenge, molecular orientation under field exposure, and revealed that a nonoriented field can induce a meaningful stereochemical bias as the dipole–field interactions are distinct for each isomer. The incorporation of statistical weighting schemes, including Boltzmann and von Mises–Fisher distributions, enabled a nuanced assessment of field–dipole alignment, including induced torque. These findings reinforce the concept that stereoselectivity can be actively changed via field–dipole coupling, with torque and polarization effects contributing to conformational rearrangements even in the absence of a directional bias.

Altogether, this study provides theoretical validation and also mechanistic insights and practical guidelines for future experimental exploration of electric field-mediated stereocontrol. The integration of directional, surface-anchored, and isotropic field models delivers a robust and adaptable framework for translating computational predictions to viable catalytic systems.

Looking ahead, these insights pave the way for the rational design of Claisen precursors bearing built-in local field generators, such as polarizable push–pull moieties or strategically placed charged substituents, that can induce the same dipole–field coupling without external instrumentation. By embedding intrinsic electric field “handles” into the substrate framework, one could program stereochemical outcomes at the molecular level, effectively turning each rearrangement precursor into its own invisible catalyst.

## Supplementary Material



## Notes

Energy stabilization for the Hurd-Nit transition states, global RMSD values, statistical weights of all substituents in both rearrangement pathways, isotropic field, and fragmentation scheme (PDF)

## References

[1] Tiago C. A. F. R., Wender A. S., Angelo H. L. M. (2015). Recent Advances in the Asymmetric
Claisen Rearrangement Promoted by Chiral Organometallic Lewis Acids
or Organic Bronsted-Lowry Acids. Curr. Org.
Synth..

[2] Lee H., Kim K. T., Kim M., Kim C. (2022). Recent Advances in
Catalytic [3,3]-Sigmatropic Rearrangements. Catalysts.

[3] Martín
Castro A. M. (2004). Claisen Rearrangement over the Past Nine Decades. Chem. Rev..

[4] Hiersemann, M. ; Nubbemeyer, U. The Claisen Rearrangement: Methods and Applications; John Wiley & Sons, 2007.

[5] Majumdar K., Nandi R. K. (2013). The Claisen rearrangement in the
syntheses of bioactive
natural products. Tetrahedron.

[6] Srikrishna A., Kumar P. P. (2000). Claisen rearrangement
based methodology for the spiroannulation
of a cyclopentane ring. Formal total synthesis of (±)-acorone
and isoacorones. Tetrahedron.

[7] Jiang S.-W., Qi X., Deng H., Gao Y., Yuan Y., Dang X., Xu B., Ma S., Xie T., Ye X.-Y., Hui Z. (2023). Design, synthesis
and anti-tumor efficacy evaluation of novel 1,3-diaryl propane-based
polyphenols obtained from Claisen rearrangement reaction. Bioorg. Chem..

[8] Anugu R. R., Mainkar P. S., Sridhar B., Chandrasekhar S. (2016). The Ireland-Claisen
rearrangement strategy towards the synthesis of the schizophrenia
drug, (+)-asenapine. Org. Biomol. Chem..

[9] Tena A., Rangou S., Shishatskiy S., Filiz V., Abetz V. (2016). Claisen thermally
rearranged (CTR) polymers. Sci. Adv..

[10] Sydlik S. A., Swager T. M. (2013). Functional graphenic materials via
a Johnson- Claisen
rearrangement. Adv. Funct. Mater..

[11] Tokuhisa H., Koyama E., Nagawa Y., Hiratani K. (2001). Synthesis of novel
dendrimers having a thermally reactive fluorescent core, and their
thermal behavior. Chem. Commun..

[12] Silva V. S., Tolentino T. A., Rodrigues T. C. A. F., Santos F. F. M., Machado D. F. S., Silva W. A., Oliveira H. C. B. d., Machado A. H. L. (2019). Unprecedented
E-stereoselectivity on the sigmatropic Hurd-Claisen rearrangement
of Morita-Baylis-Hillman adducts: a joint experimental-theoretical
study. Org. Biomol. Chem..

[13] Faulkner D. J., Petersen M. R. (1969). A synthesis of trans-trisubstituted
olefins using the
claisen rearrangement. Tetrahedron Lett..

[14] Oliveira A. G. C., Barbosa M. R., Matias P. H. F., Silva C. A. L., Machado D. F. S., de Lira Machado A. H., Benedito de Oliveira H. C. (2024). Deciphering
Stereoselectivity in Hurd-Claisen Rearrangements: A Comprehensive
Study of Electrostatic Interactions from Shubin’s Energy Decomposition
Analysis. ChemPhysChem.

[15] Fang D., Piquemal J.-P., Liu S., Cisneros G. A. (2014). DFT-steric-based
energy decomposition analysis of intermolecular interactions. Theor. Chem. Acc..

[16] Wang Z., Danovich D., Ramanan R., Shaik S. (2018). Oriented-External Electric
Fields Create Absolute Enantioselectivity in Diels-Alder Reactions:
Importance of the Molecular Dipole Moment. J.
Am. Chem. Soc..

[17] Shaik S., Danovich D., Joy J., Wang Z., Stuyver T. (2020). Electric-Field
Mediated Chemistry: Uncovering and Exploiting the Potential of (Oriented)
Electric Fields to Exert Chemical Catalysis and Reaction Control. J. Am. Chem. Soc..

[18] Mattioli E. J., Bottoni A., Zerbetto F., Calvaresi M. (2019). Oriented external
electric fields affect rate and stereoselectivity of electrocyclic
reactions. J. Phys. Chem. C.

[19] Liu F., Liu M. (2020). An Intuitive Electric-field
Contribution Decomposition Model for
Chemical Processes and Its Applications on Diels-Alder Reactions. Chem. Res. Chin. Univ..

[20] Wu X., Ren D., Wu L. (2022). Analysis of geometric structure,
electronic charge
density and absorption spectrum of germanium sulfide (GeS) under an
external electric field: A DFT calculation. Int. J. Electrochem. Sci..

[21] Dutta
Dubey K., Stuyver T., Kalita S., Shaik S. (2020). Solvent Organization
and Rate Regulation of a Menshutkin Reaction by Oriented External
Electric Fields are Revealed by Combined MD and QM/MM Calculations. J. Am. Chem. Soc..

[22] Gorin C. F., Beh E. S., Kanan M. W. (2012). An Electric Field-Induced Change
in the Selectivity of a Metal Oxide-Catalyzed Epoxide Rearrangement. J. Am. Chem. Soc..

[23] Gorin C. F., Beh E. S., Bui Q. M., Dick G. R., Kanan M. W. (2013). Interfacial
Electric Field Effects on a Carbene Reaction Catalyzed by Rh Porphyrins. J. Am. Chem. Soc..

[24] Robertson J. C., Coote M., Bissember A. (2019). Synthetic applications of light,
electricity, mechanical force and flow. Nat.
Rev. Chem..

[25] Lin Z.-R., Zeng X.-A., Yu S.-J., Sun D.-W. (2012). Enhancement of Ethanol-Acetic
Acid Esterification Under Room Temperature and Non-catalytic Condition
via Pulsed Electric Field Application. Food
Bioprocess Technol..

[26] Han Y., Lu K., Zhang Q., Jiao T., Gao Q. (2025). Density functional
theory study of the effect of external electric field on the degradation
of norfloxacin in water by hydroxyl radicals. J. Water Process Eng..

[27] Ma X., Wang Z. (2022). Removal of ciprofloxacin from wastewater by ultrasound/electric
field/sodium
persulfate (US/E/PS). Processes.

[28] Huang X., Tang C., Li J., Chen L.-C., Zheng J., Zhang P., Le J., Li R., Li X., Liu J. (2019). Electric field-induced
selective catalysis of single-molecule
reaction. Sci. Adv..

[29] Tan Z., Dong B., Xing M., Sun X., Xi B., Dai W., He C., Luo Y., Huang Y. (2024). Electric field applications
enhance the electron transfer capacity of dissolved organic matter
in sludge compost. Environ. Technol..

[30] Akamatsu M., Sakai N., Matile S. (2017). Electric-field-assisted
anion- *π* catalysis. J.
Am. Chem. Soc..

[31] Wang X., Wang H., Zheng L., Zhu C., Liang J.-X. (2021). Oriented
external electric fields regulating the oxidation reaction of CH catalyzed
by Mn-corrolazine. Int. J. Quantum Chem..

[32] Shi M. W., Thomas S. P., Hathwar V. R., Edwards A. J., Piltz R. O., Jayatilaka D., Koutsantonis G. A., Overgaard J., Nishibori E., Iversen B. B., Spackman M. A. (2019). Measurement of electric
fields experienced by urea guest molecules in the 18-Crown-6/urea
(1:5) host-guest complex: An experimental reference point for electric-field-assisted
catalysis. J. Am. Chem. Soc..

[33] Joy J., Stuyver T., Shaik S. (2020). Oriented external
electric fields
and ionic additives elicit catalysis and mechanistic crossover in
oxidative addition reactions. J. Am. Chem. Soc..

[34] Alvarado O., Alvarado M., Medina F. E., Cabrera-Pardo J. R. (2025). Effect
of oriented external electric fields on the Staudinger [2+ 2] cycloaddition
reaction: a computational study. Theor. Chem.
Acc..

[35] Aragonès A. C., Haworth N. L., Darwish N., Ciampi S., Mannix E. J., Wallace G. G., Diez-Perez I., Coote M. L. (2016). Electrostatic catalysis
of a Diels-Alder reaction. Nature.

[36] Shaik S., Ramanan R., Danovich D., Mandal D. (2018). Structure and reactivity/selectivity
control by oriented-external electric fields. Chem. Soc. Rev..

[37] Shaik S., Mandal D., Ramanan R. (2016). Oriented electric fields as future
smart reagents in chemistry. Nat. Chem..

[38] Peng Q., Duarte F., Paton R. S. (2016). Computing
organic stereoselectivity
- from concepts to quantitative calculations and predictions. Chem. Soc. Rev..

[39] Shaik S. (2024). My Vision
of Electric-Field-Aided Chemistry in 2050. ACS
Phys. Chem. Au.

[40] Ramanan R., Danovich D., Mandal D., Shaik S. (2018). Catalysis of Methyl
Transfer Reactions by Oriented External Electric Fields: Are Gold-Thiolate
Linkers Innocent?. J. Am. Chem. Soc..

[41] Luchini, G. ; Alegre-Requena, J. ; Funes-Ardoiz, I. ; Paton, R. GoodVibes: automated thermochemistry for heterogeneous computational chemistry data [version 1; peer review: 2 approved with reservations] F1000Research 2020; Vol. 9.

[42] Frisch, M. J. Gaussiañ16, revision C.01. Gaussian Inc.: Wallingford CT, 2016.

[43] Meir R., Chen H., Lai W., Shaik S. (2010). Oriented electric fields
accelerate Diels-Alder reactions and control the endo/exo selectivity. ChemPhysChem.

[44] Wang C., Danovich D., Chen H., Shaik S. (2019). Oriented External Electric
Fields: Tweezers and Catalysts for Reactivity in Halogen-Bond Complexes. J. Am. Chem. Soc..

[45] Truhlar D. G., Hase W. L., Hynes J. T. (1983). Current status of transition-state
theory. J. Phys. Chem. A.

[46] Bloomfield V. A. (1977). Hydrodynamics
in Biophysical Chemistry. Annu. Rev. Phys. Chem..

[47] Kurz, G. ; Hanebeck, U. D. Stochastic Sampling of the Hyperspherical Von Mises-fisher Distribution without Rejection Methods, 2015 Sensor Data Fusion: Trends, Solutions, Applications (SDF); 2015; pp 1–6.

[48] Fisher R. A. (1953). Dispersion
on a sphere. Proc. R. Soc. London, Ser. A.

