# Supplementary material for: Unveiling Electric Field-Driven Stereocontrol in Hurd–Claisen Rearrangements
Source: J Chem Theory Comput. 2026 Jun 25;22(21):11419–31. doi: 10.1021/acs.jctc.6c00732 (PMC13757751; doi:10.1021/acs.jctc.6c00732)
Supplement: Supplementary file 1 [file ct6c00732_si_001.pdf]

Unveiling Electric-Field-Driven Stereocontrol in Hurd–Claisen Rearrangements

Mateus Rodrigues Barbosa,[†] Pedro H. F. Matias,[†] Daniel F. Scalabrini Machado,[‡] Angelo H. Lira Machado,[¶] and Heibbe C. B. de Oliveira[*,†]

†*Laboratório de Estrutura Eletrônica e Dinâmica Molecular, Universidade Federal de Goiás, Goiânia 74690-900, GO, Brasil.*

‡*Laboratório de Modelagem de Sistemas Complexos, Institute of Chemistry, Universidade de Brasília, Brasília, DF 70910-900, Brasil*

¶*Laboratório de Isolamento e Transformação de Moléculas Orgânicas, Institute of Chemistry, Universidade de Brasília, Brasília, DF 70910-900, Brasil*

E-mail: heibbe@ufg.br

Sumário

Reaction Axis Energy Stabilization **2**

Reaction Axis Global RMSD **5**

Statistical Weights **6**

Isotropic Field **9**

Fragmentation Scheme **14**

Reaction Axis Energy Stabilization

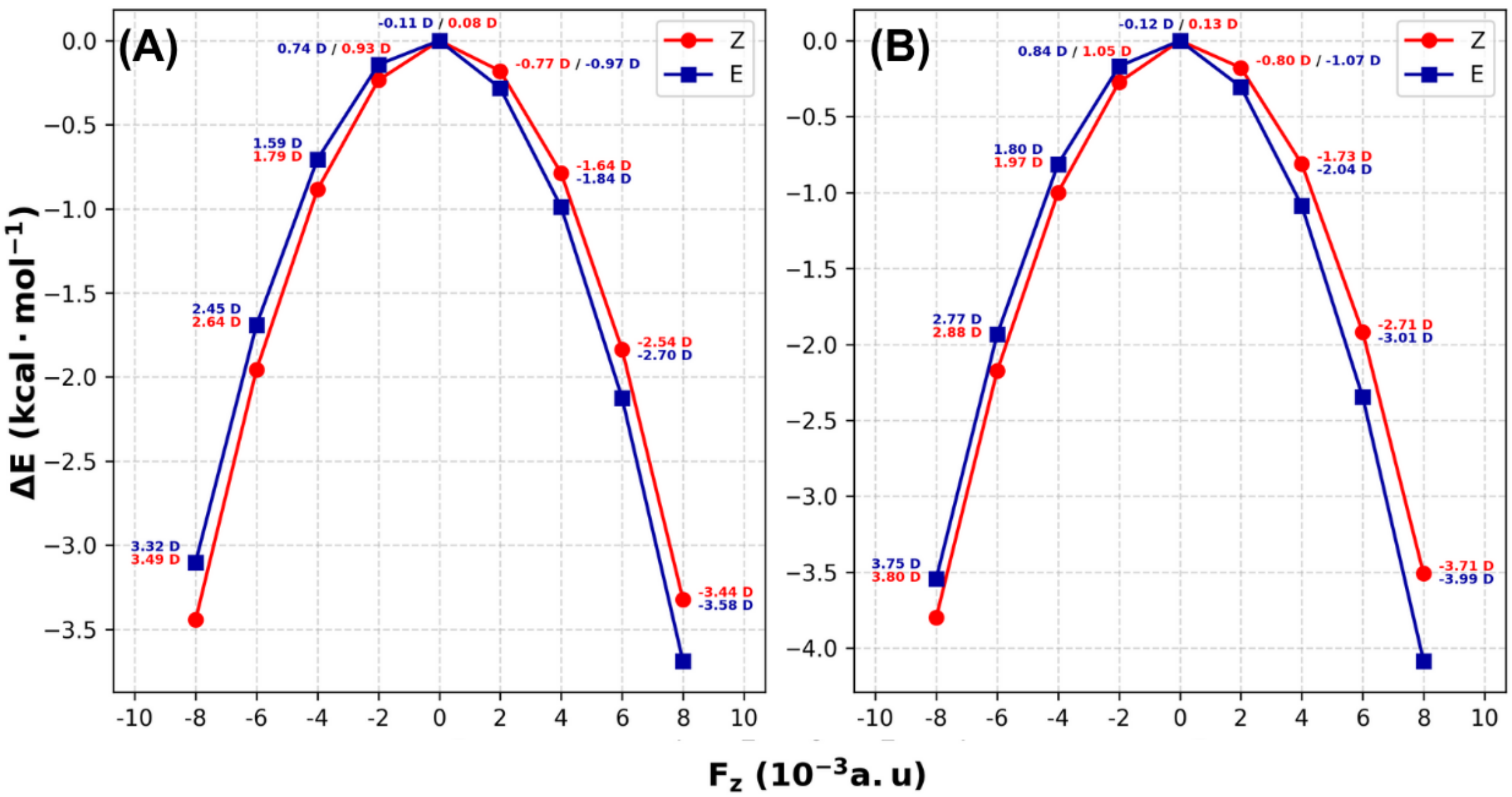

Figure S1: Relative electronic energy differences (with respect to $F_z = 0$) of transition states for the hurd-est rearrangement of (A) Et and (B) SProp substituents. $\Delta\Delta G^{\ddagger}$ were calculated using Grimme's *quasi*-harmonic approximation at 373 K with the M06-2X/def2-TZVPP//M06-2X/6-311+G(d,p) level of theory.

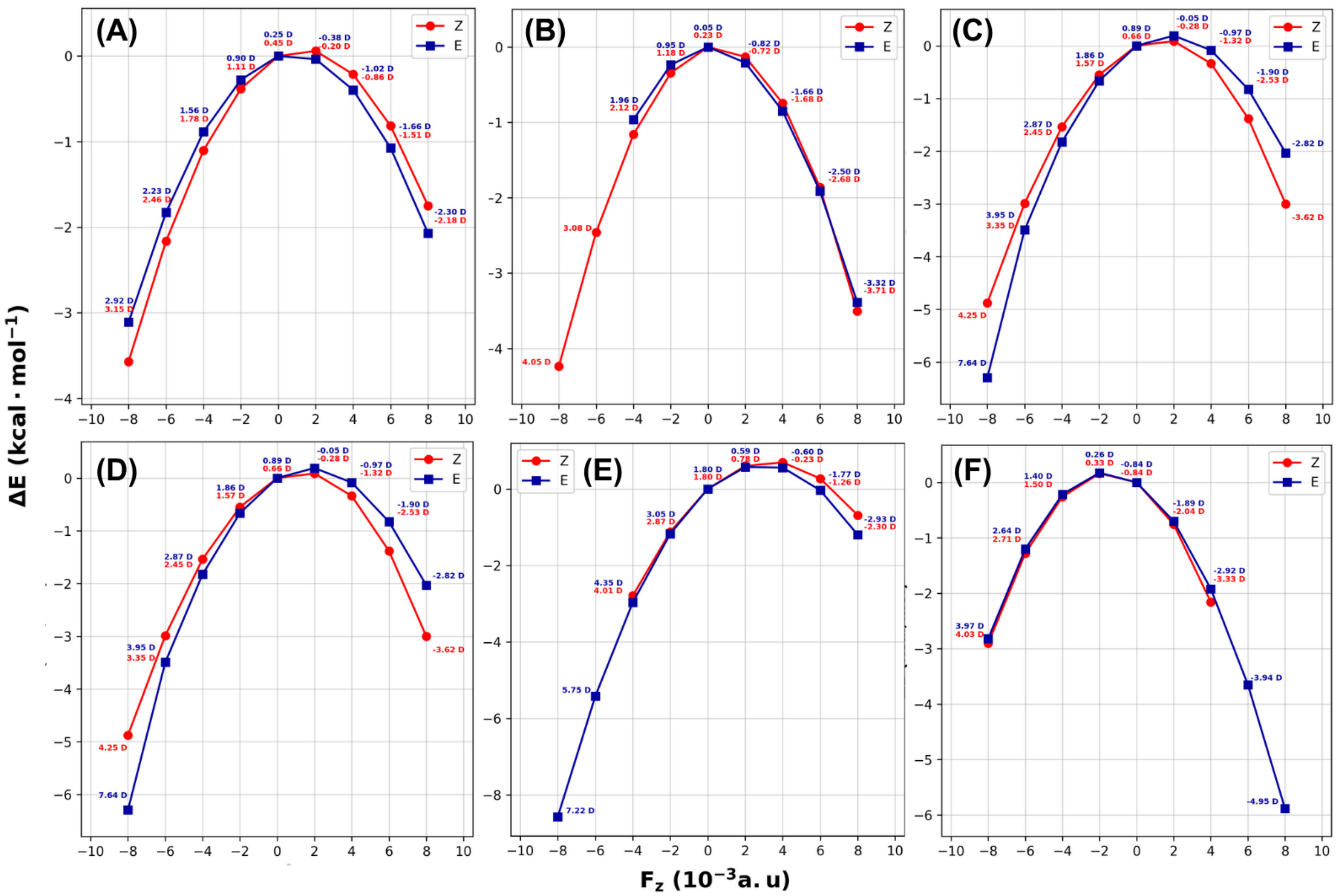

Figure S2: Relative electronic energy differences (with respect to $F_z = 0$) of transition states for the hurd-nit rearrangement of (A) CH3, (B) IsoBut, (C) TBut, (D) Ph, (E) NO_2Ph and (F) NH_2Ph substituents. $\Delta\Delta G^{\ddagger}$ were calculated using Grimme's *quasi*-harmonic approximation at 373 K with the M06-2X/def2-TZVPP//M06-2X/6-311+G(d,p) level of theory.

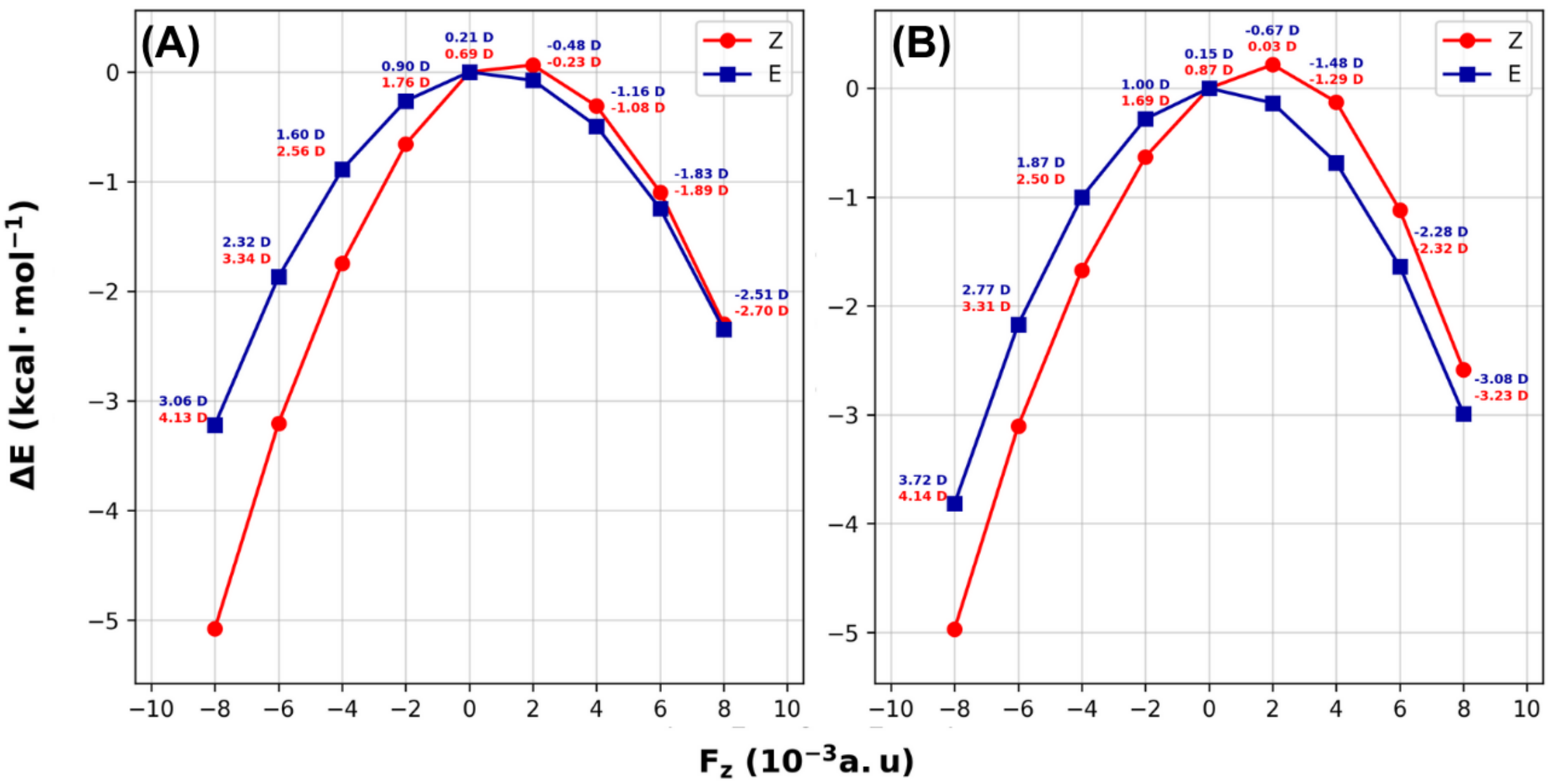

Figure S3: Relative electronic energy differences (with respect to $F_z = 0$) of transition states for the hurd-nit rearrangement of (A) Et and (B) SProp substituents. $\Delta\Delta G^{\ddagger}$ were calculated using Grimme's *quasi*-harmonic approximation at 373 K with the M06-2X/def2-TZVPP//M06-2X/6-311+G(d,p) level of theory.

Reaction Axis Global RMSD

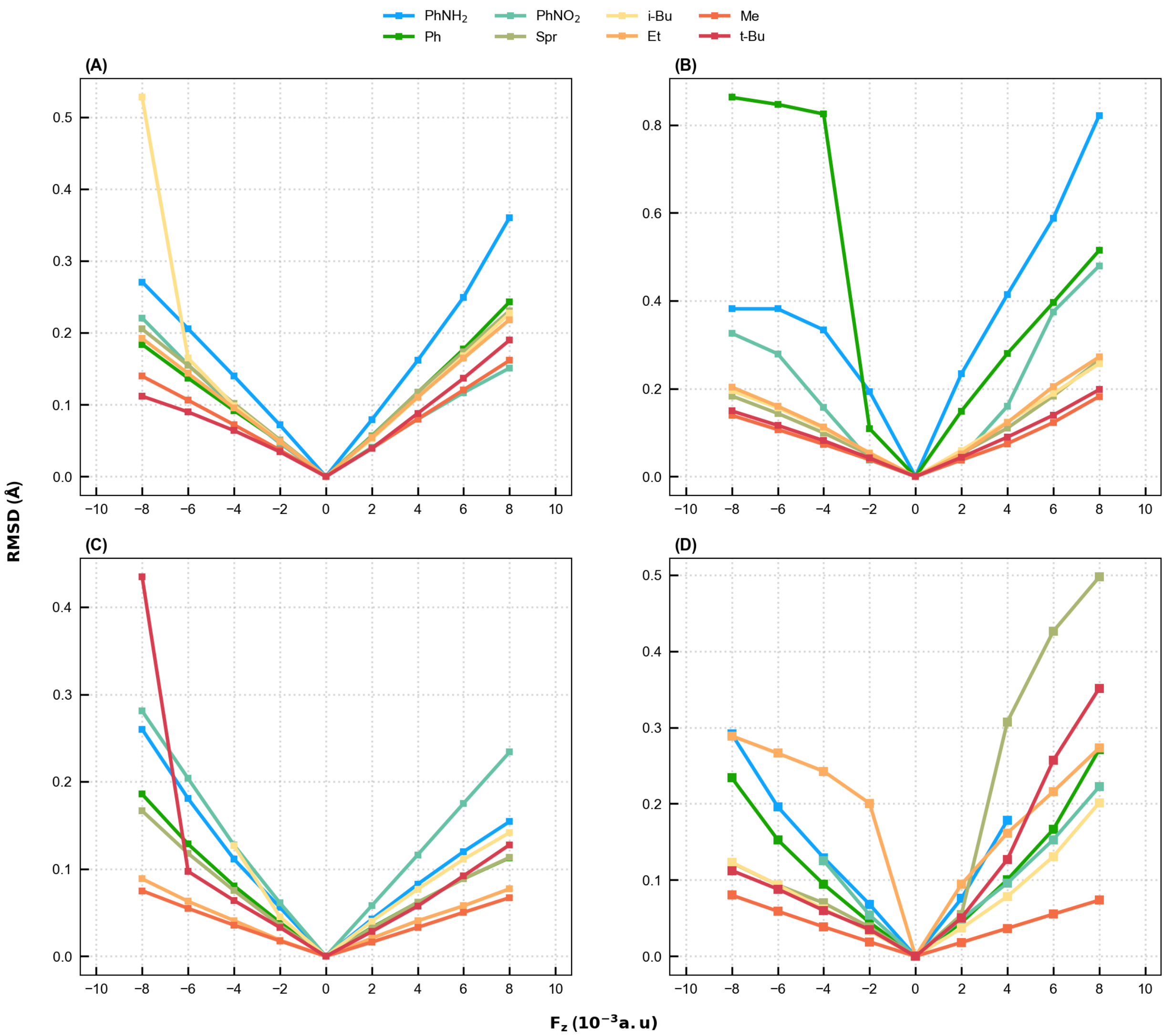

Figure S4: RMSD of the (A) E- and (B) Z-isomers for the Hurd-Est rearrangement transition state, and the (C) E- and (D) Z-isomers for the Hurd-Nit rearrangement, as a function of the OEEF. The extremely high RMSD values for some of the bulkier molecules are due to the fact that the OEEF is not able to properly sample the conformational space.

Statistical Weights

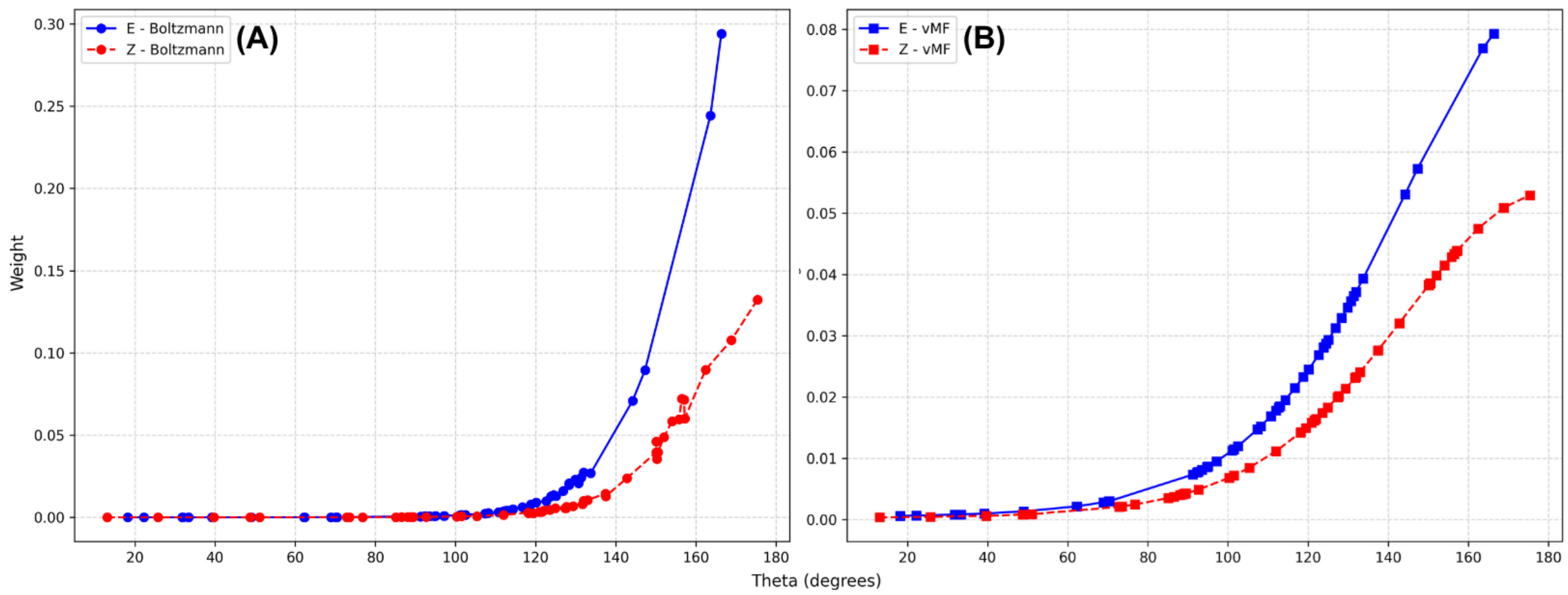

Figure S5: Statistical Weights of the CH_3 Hurd-Est rearrangment under 50 randomly oriented eletric fields (0.004 a.u), comparing Boltzmann (left) and von Mises-Fischer (right) distributions. Color coding: E (blue), Z-isomer (red).

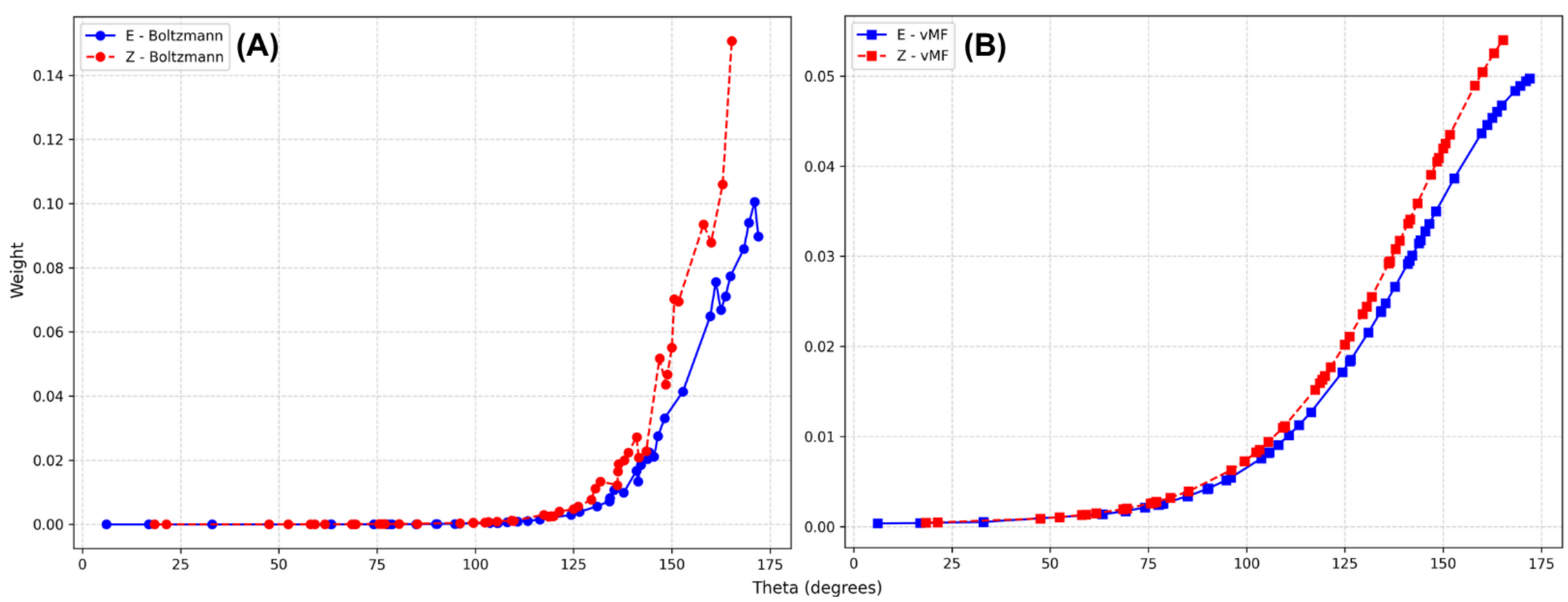

Figure S6: Statistical Weights of the *SProp* Hurd-Est rearrangment under 50 randomly oriented eletric fields (0.004 a.u), comparing Boltzmann (left) and von Mises-Fischer (right) distributions. Color coding: E (blue), Z-isomer (red).

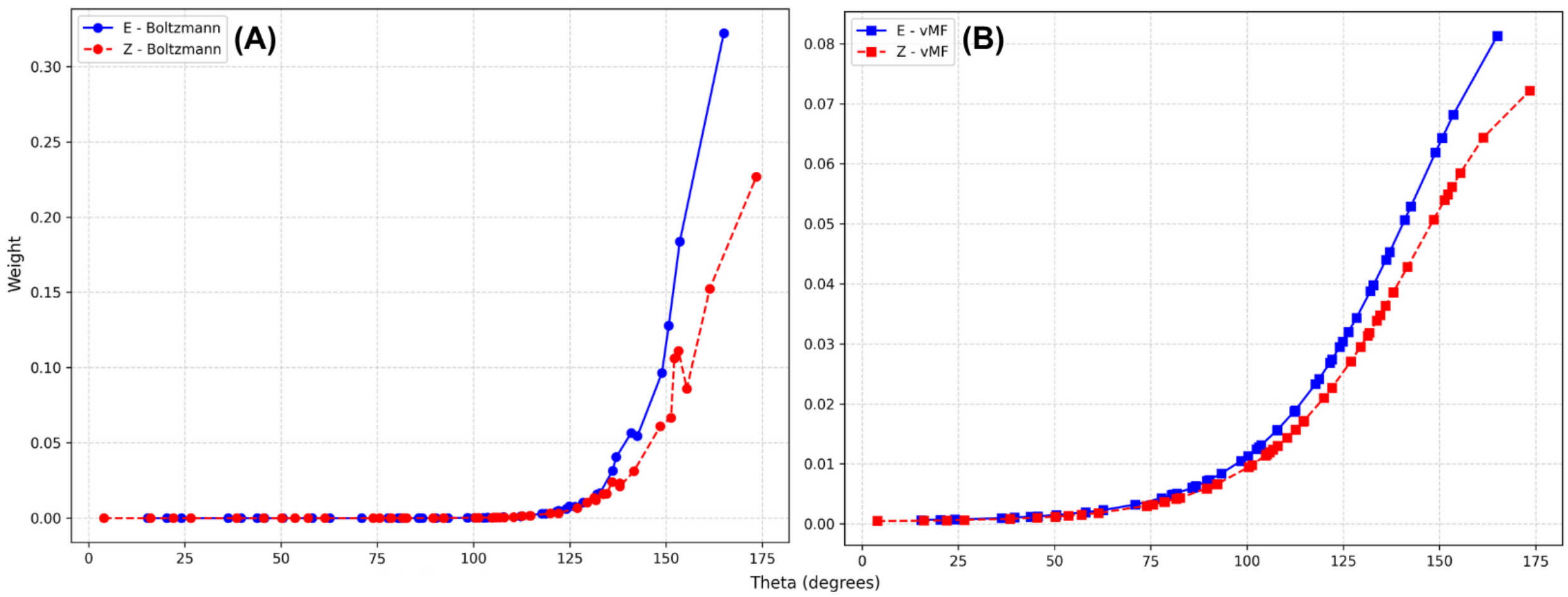

Figure S7: Statistical Weights of the CH_3 Hurd-Nit rearrangment under 50 randomly oriented eletric fields (0.004 a.u), comparing Boltzmann (left) and von Mises-Fischer (right) distributions. Color coding: E (blue), Z-isomer (red).

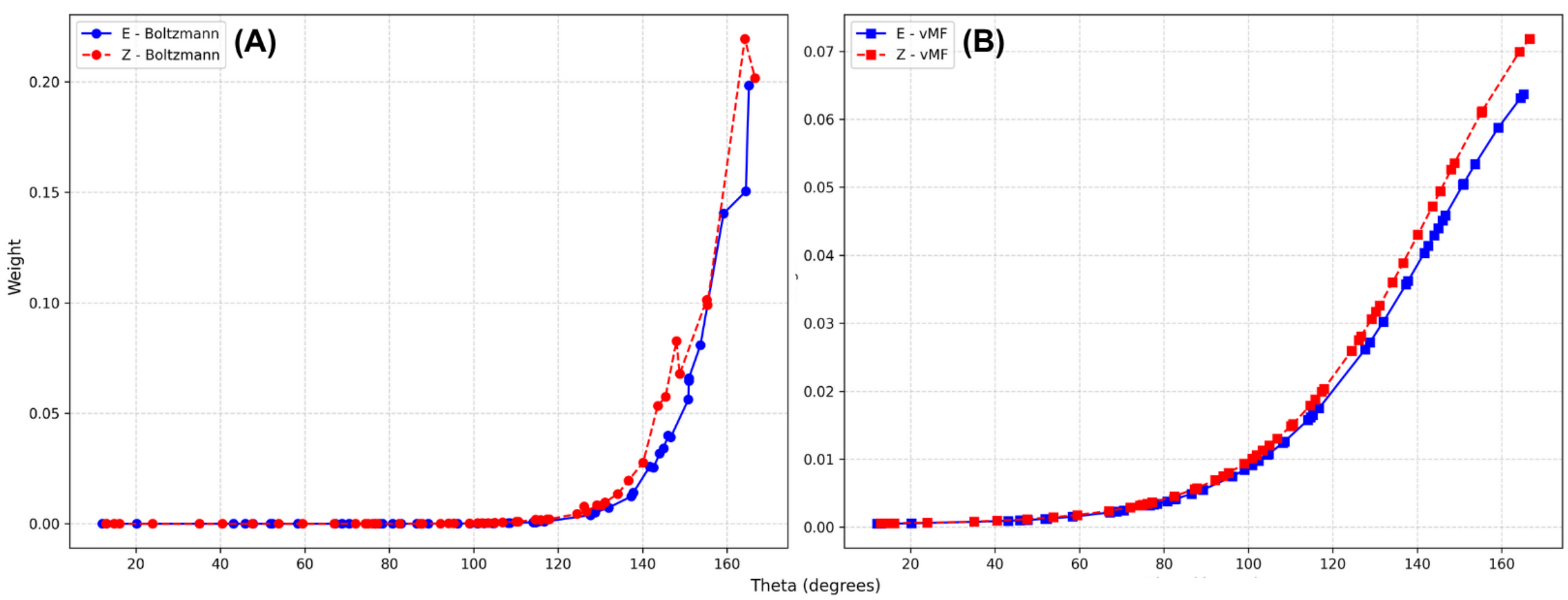

Figure S8: Statistical Weights of the Et Hurd-Nit rearrangment under 50 randomly oriented eletric fields (0.004 a.u), comparing Boltzmann (left) and von Mises-Fischer (right) distributions. Color coding: E (blue), Z-isomer (red).

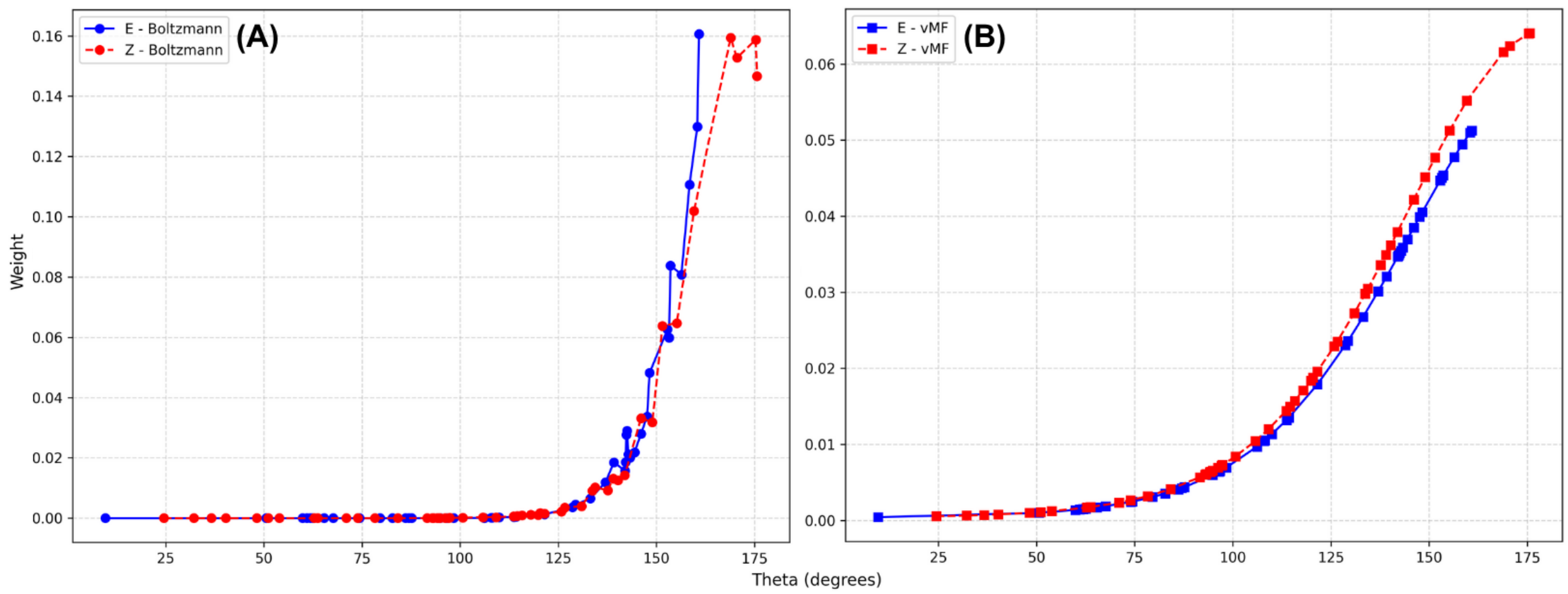

Figure S9: Statistical Weights of the *Ph* Hurd-Nit rearrangment under 50 randomly oriented eletric fields (0.004 a.u), comparing Boltzmann (left) and von Mises-Fischer (right) distributions. Color coding: E (blue), Z-isomer (red).

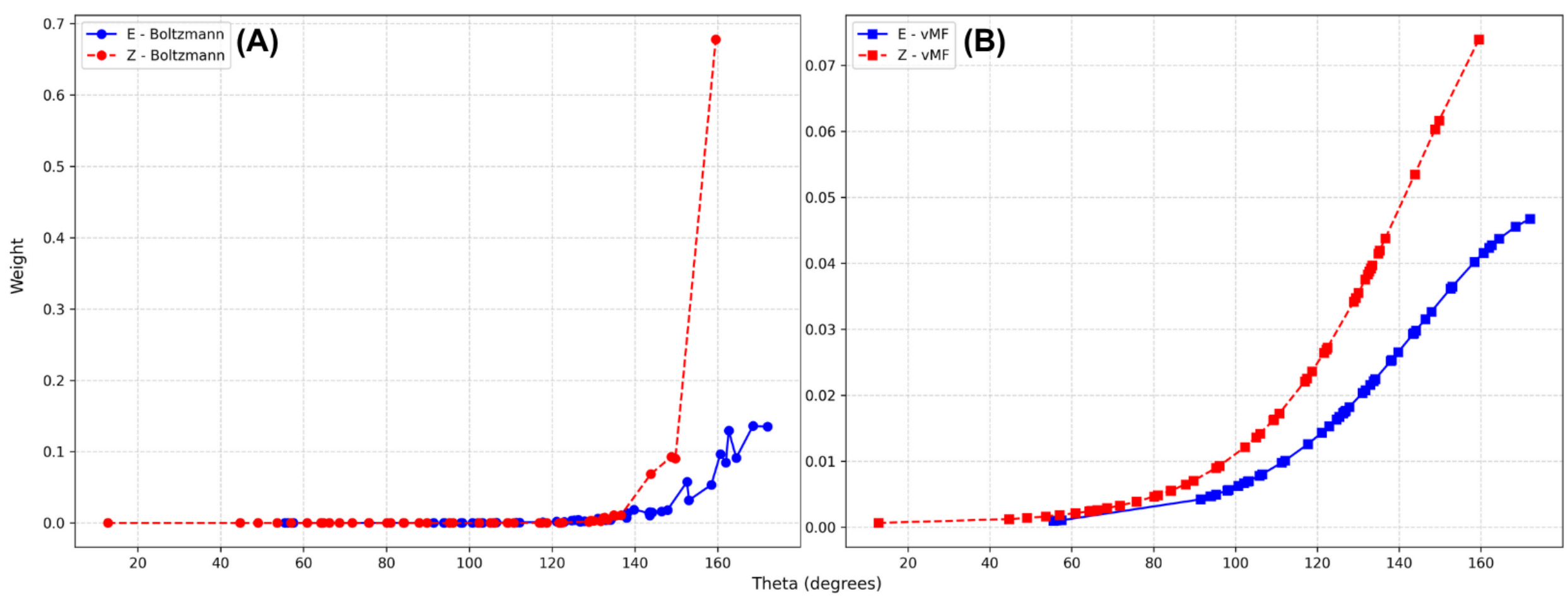

Figure S10: Statistical Weights of the NO_2Ph Hurd-Nit rearrangment under 50 randomly oriented eletric fields (0.004 a.u), comparing Boltzmann (left) and von Mises-Fischer (right) distributions. Color coding: E (blue), Z-isomer (red).

Isotropic Field

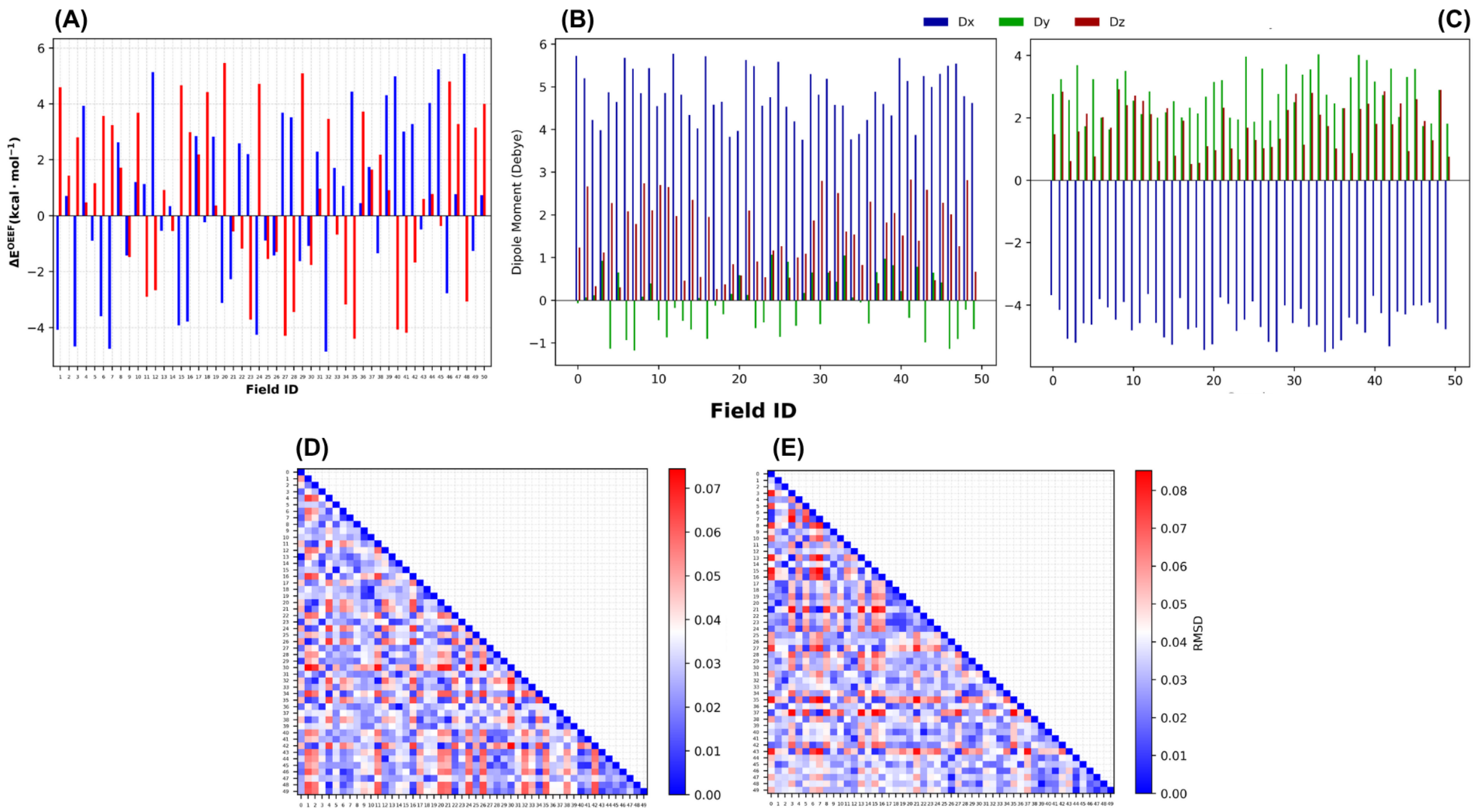

Figure S11: Field-induced perturbations in the CH_3 Hurd-Nit rearrangement under isotropic OEEFs (0.004 a.u.). (A) Transition state energy changes for E- (blue) and Z- (red) isomers across 50 randomized field orientations (numbered 1-50). (B,C) Corresponding dipole moment components (x, y, z) for the *Z*- and *E*-isomers, respectively. (D,E) Root-mean-square deviation (RMSD) of the *E*- and *Z*-isomers, respectively. Note the different scales of the RMSD values for each isomer.

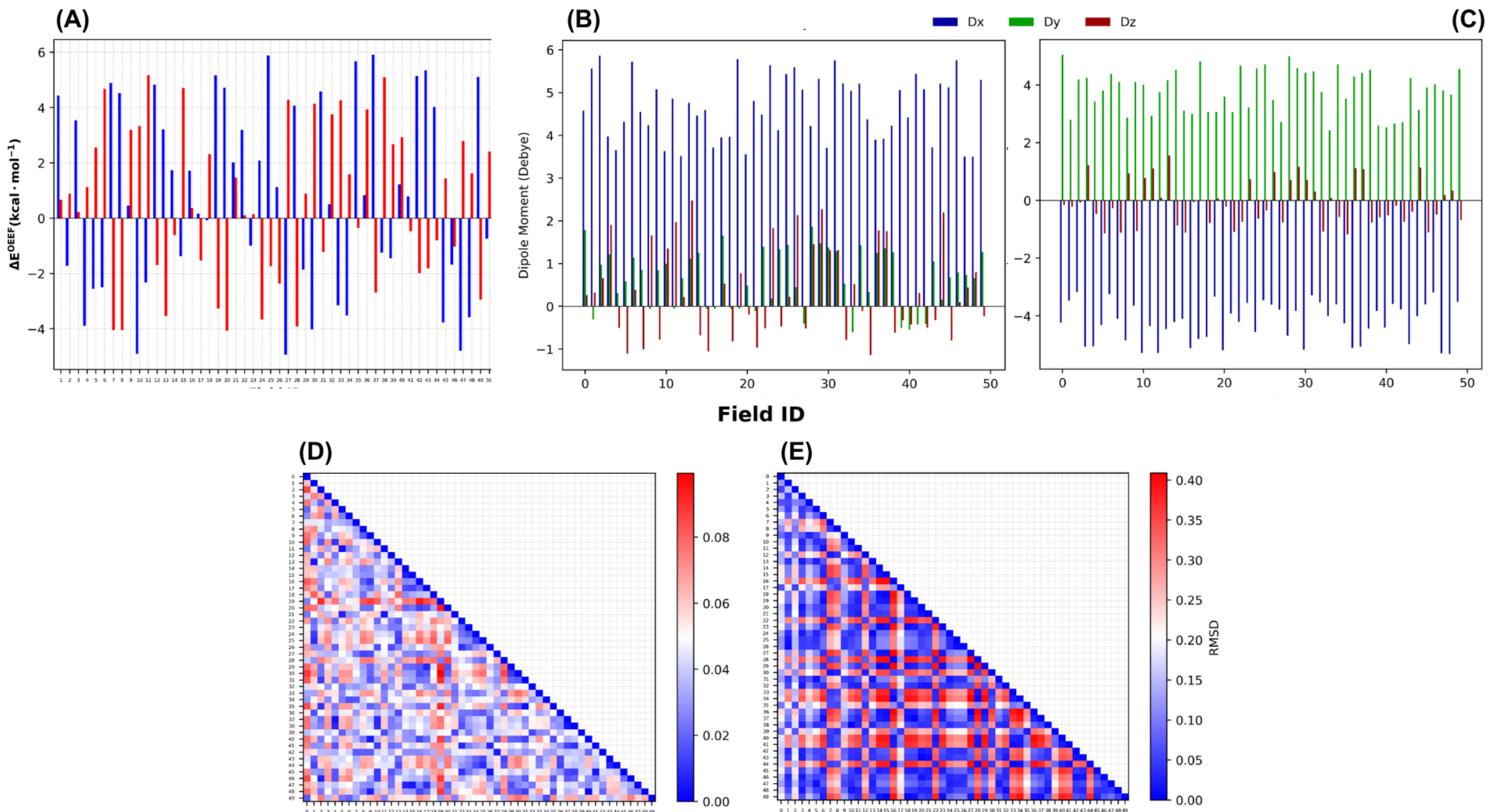

Figure S12: Field-induced perturbations in the Et Hurd-Nit rearrangement under isotropic OEEFs (0.004 a.u.). (A) Transition state energy changes for E- (blue) and Z- (red) isomers across 50 randomized field orientations (numbered 1-50). (B,C) Corresponding dipole moment components (x, y, z) for the *Z*- and *E*-isomers, respectively. (D,E) Root-mean-square deviation (RMSD) of the *E*- and *Z*-isomers, respectively. Note the different scales of the RMSD values for each isomer.

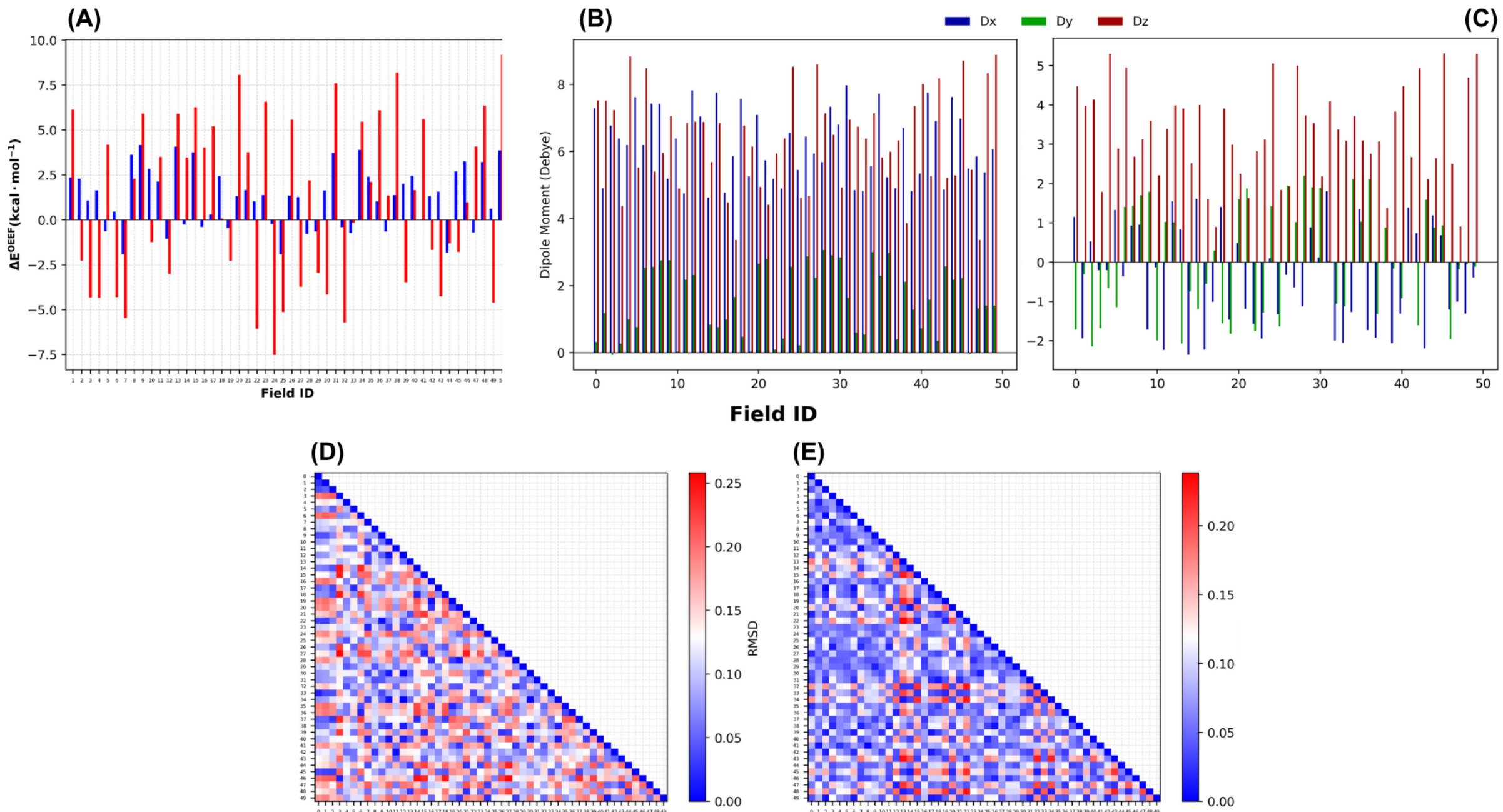

Figure S13: Field-induced perturbations in the NO2Ph Hurd-Nit rearrangement under isotropic OEEFs (0.004 a.u.). (A) Transition state energy changes for E- (blue) and Z- (red) isomers across 50 randomized field orientations (numbered 1-50). (B,C) Corresponding dipole moment components (x, y, z) for the *Z*- and *E*-isomers, respectively. (D,E) Root-mean-square deviation (RMSD) of the *E*- and *Z*-isomers, respectively. Note the different scales of the RMSD values for each isomer.

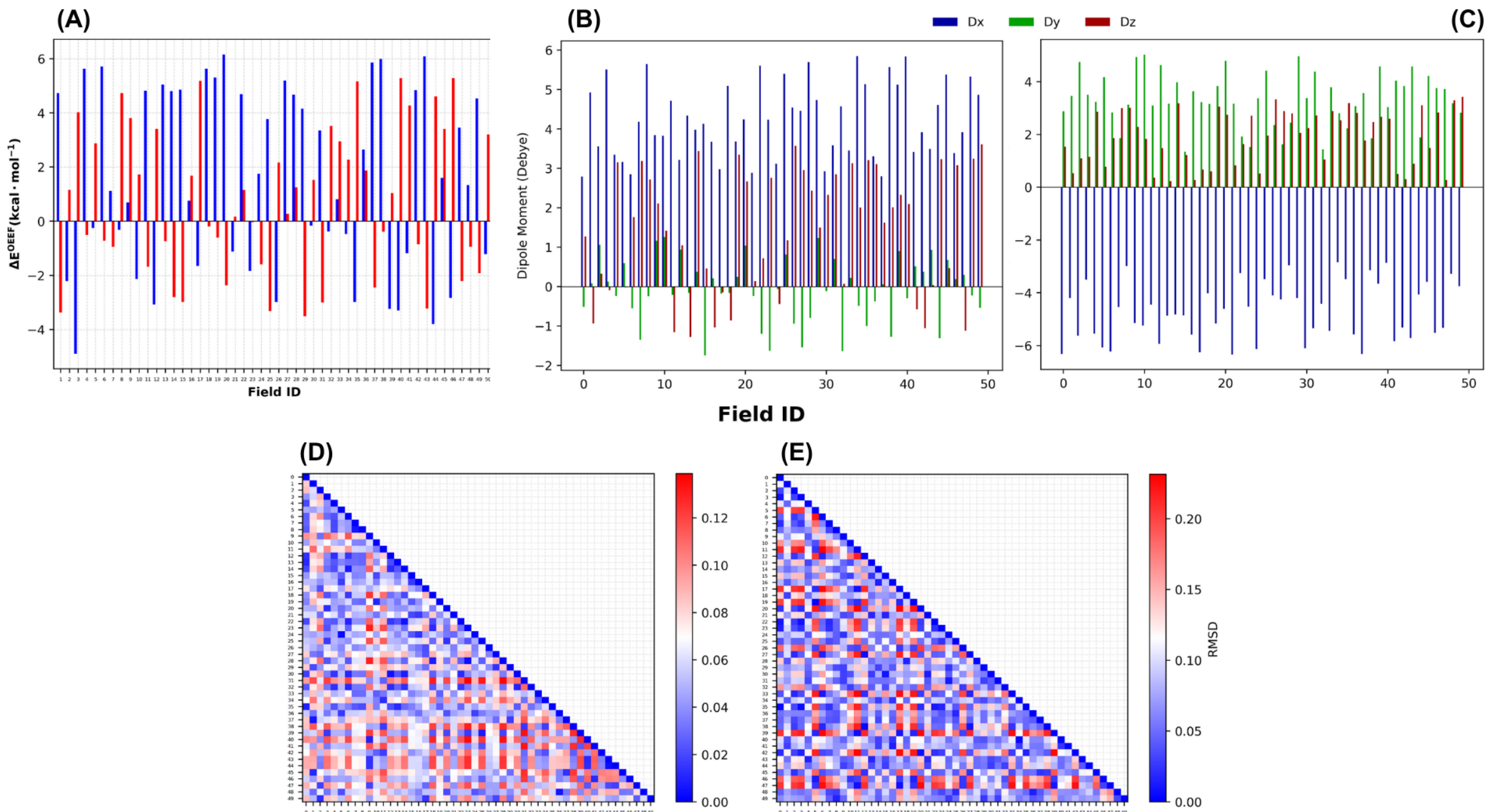

Figure S14: Field-induced perturbations in the Ph Hurd-Nit rearrangement under isotropic OEEFs (0.004 a.u.). (A) Transition state energy changes for E- (blue) and Z- (red) isomers across 50 randomized field orientations (numbered 1-50). (B,C) Corresponding dipole moment components (x, y, z) for the *Z*- and *E*-isomers, respectively. (D,E) Root-mean-square deviation(RMSD) of the *E*- and *Z*-isomers, respectively. Note the different scales of the RMSD values for each isomer.

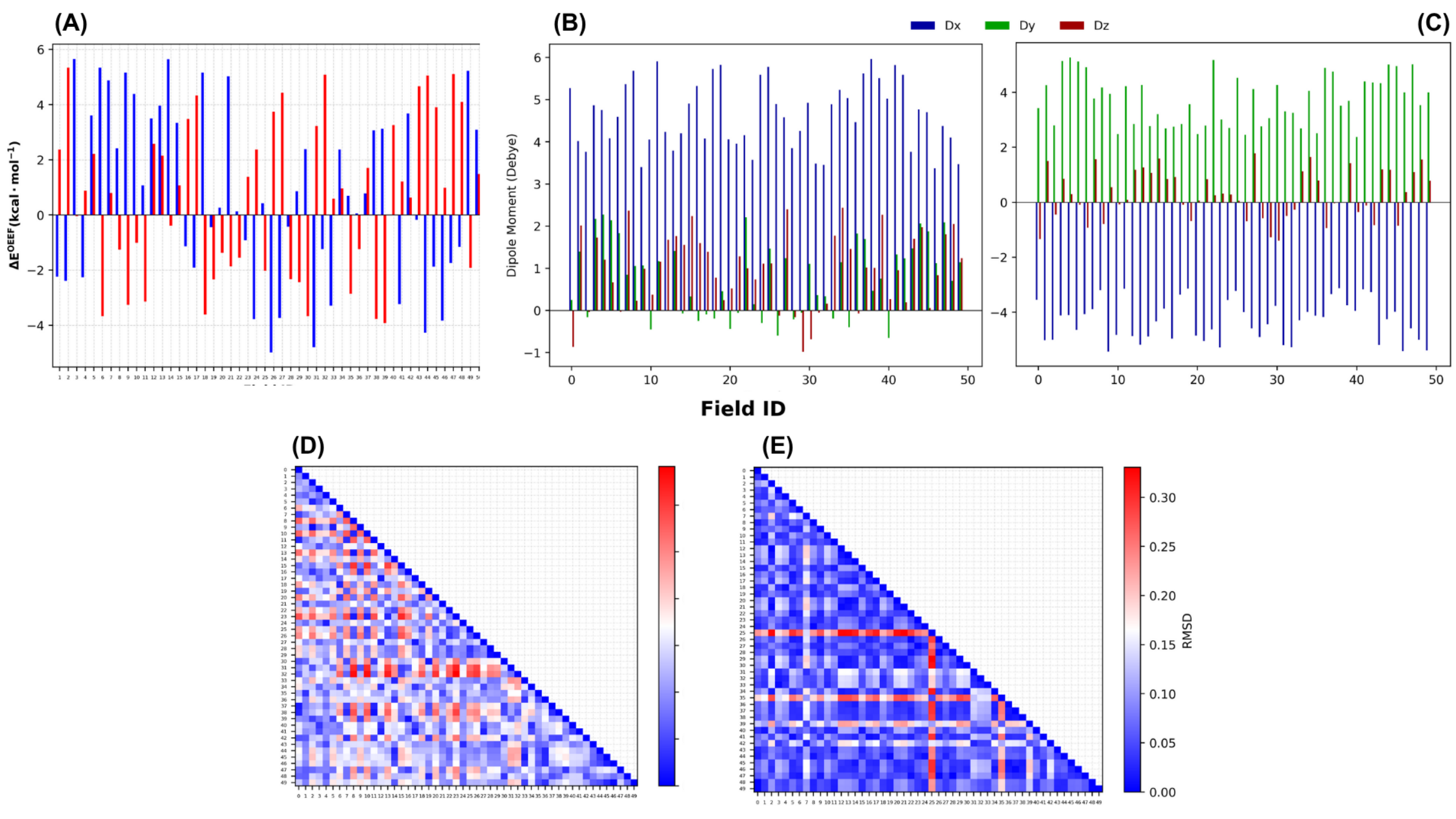

Figure S15: Field-induced perturbations in the SProp Hurd-Nit rearrangement under isotropic OEEFs (0.004 a.u.). (A) Transition state energy changes for E- (blue) and Z- (red) isomers across 50 randomized field orientations (numbered 1-50). (B,C) Corresponding dipole moment components (x, y, z) for the *Z*- and *E*-isomers, respectively. (D,E) Root-mean-square deviation (RMSD) of the *E*- and *Z*-isomers, respectively. Note the different scales of the RMSD values for each isomer.

Fragmentation Scheme

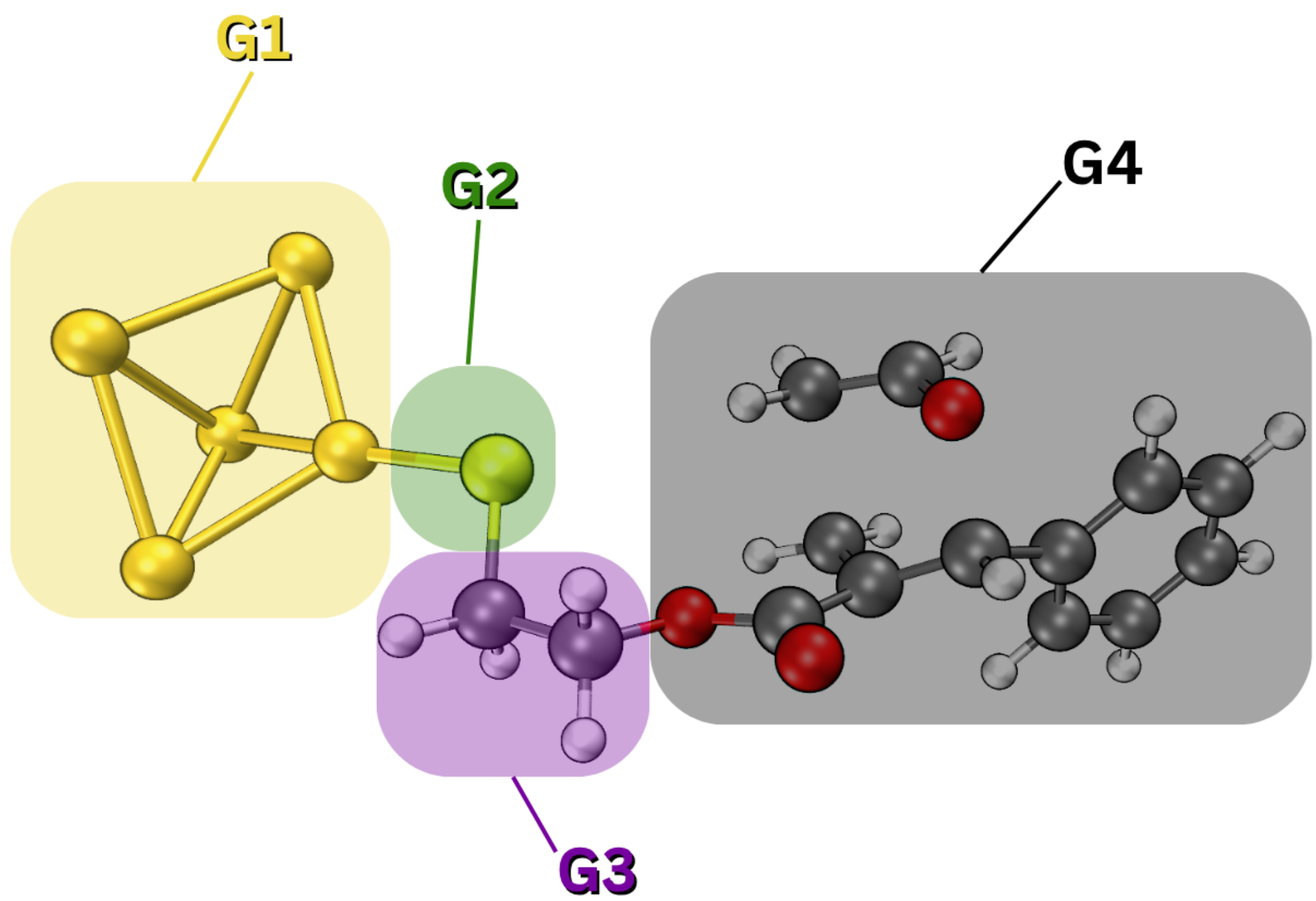

Figure S16: Molecular fragmentation scheme used to compute Mulliken group charges. The fragments G1–G4 were defined to enable a detailed analysis of charge redistribution under varying orientations and strengths of oriented external electric fields (OEEFs).
